# Cell-Free Immune-Conditioned Products in Advanced Solid Tumors: A Source-Aware Translational Framework

**DOI:** 10.3390/medicina62101964

**Published:** 2026-10-11

**Authors:** Ramón Gutiérrez-Sandoval, Francisco Gutiérrez-Castro, Natalia Muñoz-Godoy, Ider Rivadeneira, Andy Lagos, Jordan Iturra, Francisco Krakowiak, Ignacio Muñoz, Cristián Peña-Vargas, Andrés Toledo

**Affiliations:** 1 OGRD Alliance LLC, 6900 Tavistock Lakes Boulevard, Suite 400, Orlando, FL 32827, USA; sourcing@ogrdalliance.org (C.P.-V.); ops@ogrdalliance.org (A.T.); 2 Department of Cancer Research, Flowinmunocell-Bioexocell Group, 08028 Barcelona, Spain; servicios@flowinmunocell.cl (F.G.-C.); contacto@flowinmunocell.cl (N.M.-G.); 3 Department of Outreach and Engagement Programs, OGRD Consortium, Charlestown KN0802, Saint Kitts and Nevis; iderlautaro@gmail.com (I.R.); lagosandy@gmail.com (A.L.); jiconsultant@ogrdconsorcio.com (J.I.); kinesiologo@recell.cl (I.M.); 4 Histopathology Laboratory, Bioclas, Concepción 4030000, Chile; fkconsultant@ogrdconsorcio.com

**Keywords:** advanced solid tumors, cancer immunotherapy, cell-free immune-conditioned products, extracellular lipid–protein matrices, phospholipoproteomic matrix, membrane microdomains, microdomain reconstruction, intradermal administration, new approach methodologies, translational oncology

## Abstract

*Background and Objectives*: Cell-free immune-conditioned products represent an emerging class of complex biological preparations with potential relevance to cancer immunotherapy. Their translation requires clear source definition, reproducible product characterization, mechanism-linked testing, and prospective clinical evaluation. This review aimed to develop a source-aware translational framework for their assessment in advanced solid tumors. *Materials and Methods*: PubMed/MEDLINE, consensus statements, professional-society documents, regulatory resources, and reporting guidelines were reviewed through 2 August 2026. Seventy-one references were integrated using a structured critical-review approach aligned with the Scale for the Assessment of Narrative Review Articles (SANRA), covering product identity, analytical quality, mechanism, delivery, human-relevant nonclinical evidence, clinical assessment, and evidence governance. *Results*: The framework separates biological source from recovered product class and organizes development through progressively stronger evidence layers spanning analytical qualification, mechanism-linked potency, human biological engagement, patient-centered assessment, tumor-level activity, and comparative validation. Mechanistic concepts such as membrane microdomain reorganization are positioned as testable intermediate evidence rather than clinical surrogates. Intradermal administration and new approach methodologies are incorporated as product-specific translational tools, while a published dendritic cell-conditioned evidence lineage illustrates how the framework can be operationalized. *Conclusions*: This source-aware framework provides an integrated and testable architecture for advancing complex cell-free immune-conditioned products from product qualification toward prospective clinical evaluation and comparative validation.

## 1. Introduction

Modern cancer immunotherapy has established that durable tumor control can be achieved by modifying the interaction between malignant tissue and host immunity rather than by relying exclusively on direct cytotoxicity. Immune checkpoint inhibitors, therapeutic vaccines, adoptive cellular strategies, and biomarker-guided combinations have consequently altered the treatment landscape. Nevertheless, durable responses remain limited by tumor heterogeneity, immune exclusion, dysfunctional antigen presentation, treatment-related immunological injury, and declining host reserve. These challenges provide a clear rationale for strategies directed at immune organization and competence, with mechanistic plausibility and clinical efficacy evaluated as distinct evidence layers.

A parallel problem arises from the populations in whom new strategies are most often considered. Oncology trials commonly favor preserved organ function, adequate performance status, limited comorbidity, and the ability to complete intensive follow-up. Patients with frailty, prior toxicities, concurrent illness, logistical barriers, or declining performance status are therefore underrepresented, even though they constitute an important proportion of advanced cancer care. A patient may consequently have active disease and a desire for further treatment but no immediately suitable standard option or accessible trial.

Cell-free immune-conditioned products have emerged within this context. Their administered material may contain extracellular vesicles, soluble mediators, membrane fragments, lipid–protein assemblies, metabolites, or mixed fractions generated under defined culture and processing conditions. Adjacent source architectures—including plant-derived extracellular vesicles and manufactured phytochemical–phospholipid complexes—further illustrate why origin, composition, and process must be distinguished rather than inferring the mechanism from shared lipid–protein features [1,2]. Potential advantages of conditioned products include the absence of viable therapeutic cells, compatibility with scalable processing, repeated administration, and mechanism-linked analytical testing. Key development priorities include controlling source-dependent heterogeneity, defining active attributes, harmonizing nomenclature, ensuring manufacturing reproducibility, and prospectively linking composition to biological and clinical outcomes.

The expression “phospholipoproteomic matrix” has conceptual value but also terminological risk. In this review, it is used only as a conditional analytical descriptor for an extracellular lipid–protein architecture in which phospholipid-containing structures and proteinaceous components form reproducible, activity-linked assemblies [3]. It does not identify a brand or source, imply an extracellular vesicle subtype, or predetermine a regulatory class. Comparable lipid–protein architectures may be generated from human immune cells, other human cells, non-human biological systems, plants, microbial or fermentation systems, manufactured phytochemical–phospholipid complexes, or defined recombinant and synthetic components. These materials cannot be treated as interchangeable merely because lipids and proteins are present.

The intradermal route provides a further translational opportunity. Human skin contains specialized antigen-presenting and innate immune cell populations and drains efficiently to regional lymphatics [4]. Low-volume delivery can be performed in outpatient settings and incorporated into repeated sampling schedules [5]. Accordingly, its translational value can be established prospectively through product-specific assessment of delivery performance, tolerability, biological engagement, and tumor-level outcomes.

Existing reviews and methodological guidance have addressed individual components of this field, including extracellular vesicle nomenclature and characterization, secretome standardization, potency testing, dendritic cell immunobiology, membrane organization, intradermal immune delivery, and the regulatory use of new approach methodologies (NAMs). However, these domains have largely been treated independently. To our knowledge, no previous review has integrated biological source, recovered product class, analytical identity and potency, membrane microdomain hypotheses, intradermal administration, NAM-aligned evidence, patient selection, longitudinal clinical assessment, and claim-specific evidence thresholds within a single translational architecture for advanced solid tumors. The present review was designed to address this integration gap rather than to replace source-specific or method-specific guidance.

This review establishes a source-aware translational framework for cell-free immune-conditioned products in advanced solid tumors. The framework distinguishes source architecture from recovered product class, defines minimum analytical and potency requirements, formalizes microdomain reconstruction as a falsifiable mechanistic framework grounded in membrane microdomain and immune-synapse biology [6], positions NAM-aligned evidence within clinical development, defines a three-module clinical monitoring architecture [7], and aligns each claim with the evidence required to sustain it.

## 2. Structured Review Methods

### 2.1. Objective and Review Questions

The review asked how a complex cell-free immune-conditioned product could be developed for adults with advanced solid tumors while preserving analytical rigor, ethical proportionality, mechanistic falsifiability, and interpretable clinical evidence. Seven linked questions were specified: (i) How should the intended population be defined without creating a new diagnostic category? (ii) How should nomenclature separate source architecture from recovered product class? (iii) What analytical and potency evidence is required before clinical interpretation? (iv) How can membrane microdomains be evaluated as a mechanism rather than assumed as a property? (v) What supports intradermal delivery as a research route? (vi) How can NAMs strengthen nonclinical evidence without being conflated with clinical phases? (vii) How should immune, functional, metabolic, structural, and clinical outcomes be sequenced [8]?

### 2.2. Information Sources and Search Strategy

A structured PubMed/MEDLINE search was conducted and updated through 2 August 2026. Searches were supplemented by citation chaining and targeted retrieval from consensus, professional-society, regulatory, and reporting-guideline repositories [9]. The search covered advanced solid tumors and treatment eligibility; host-directed immunobiology; human and non-human secretomes; extracellular vesicles and mixed lipid–protein assemblies; plant-derived extracellular materials; phytochemical–phospholipid complexes; membrane microdomains, lipid rafts, immune-synapse organization, and microdomain reconstruction; product identity, purity, potency, stability, and lot comparability; intradermal delivery; NAMs and human-relevant nonclinical systems; performance status and patient-reported outcomes; Response Evaluation Criteria in Solid Tumours (RECIST), immune RECIST (iRECIST), PET Response Criteria in Solid Tumours (PERCIST), and immune-adapted imaging; real-world evidence; research ethics; and re-porting standards. Reproducible search blocks, the evidence-domain map, and the published evidence-lineage map are provided in the Supplementary Materials (Tables S1–S3). The targeted update from 24 July through 2 August 2026 did not identify a source that materially altered the predefined framework; the interpretive corpus therefore retained 71 references.

### 2.3. Eligibility, Selection, and Evidence Charting

References were included when they materially informed at least one predefined domain: clinical population definition, source or product taxonomy, analytical quality, mechanism-linked potency, intradermal delivery, NAM context of use, longitudinal outcomes, or evidence governance. Records were excluded when they were duplicative, outside the predefined translational domains, purely descriptive without decision-relevant methodological or mechanistic content, focused on unrelated disease settings without transferable relevance, or insufficiently informative to support a defined component of the framework. Selection was therefore based on relevance to predefined framework questions rather than on exhaustive enumeration of all publications within each topic. Priority was given to consensus statements, systematic or high-quality narrative reviews, regulatory documents, reporting standards, pivotal methodological publications, and representative translational studies [10]. Product-specific publications were charted as a published evidence lineage when they contributed a defined analytical, functional, monitoring, traceability, or mechanistic module. Searches and primary evidence charting were performed by R.G.-S.; eligibility and citation verification were independently reviewed by F.G.-C. and N.M.-G.; methodological, analytical, and regulatory statements were reviewed by F.K., I.M., and A.T. Disagreements were resolved by discussion. The review analyzed published evidence and publicly available regulatory documents and did not incorporate unpublished patient-level efficacy or safety data.

Because this was a structured critical review rather than a systematic review, study selection was not intended to generate a pooled or exhaustive estimate of treatment effects. The synthesis explicitly considered potential publication bias, selective availability of detailed product-characterization studies, heterogeneity across source materials and experimental systems, and author-group familiarity with the published evidence lineage. Their influence was managed by separating evidence layers, maintaining explicit claim boundaries, prioritizing external methodological and consensus sources, and independently evaluating clinical, mechanistic, and biomarker evidence.

### 2.4. Synthesis and Methodological Boundaries

The 71-reference evidence corpus was charted by domain and integrated qualitatively according to SANRA principles: justification of importance, explicit aims, a described literature search, appropriate referencing, scientific reasoning, and presentation of clinically relevant endpoints. The synthesis was designed for framework construction rather than pooled treatment-effect estimation [11]. It preserves distinct evidence layers for product identity, biological activity, route feasibility, clinical observation, and comparative validation. For quantitative transparency, the 71-reference corpus was mapped to 11 predefined evidence domains: eligibility and representativeness (*n* = 5), skin and intradermal immunobiology (*n* = 5), review methodology and host-directed rationale (*n* = 8), source and product taxonomy (*n* = 11), characterization, potency, and platform examples (*n* = 16), microdomain mechanisms and stability (*n* = 10), NAMs and nonclinical modernization (*n* = 8), function and patient-reported outcomes (*n* = 5), metabolic and structural imaging (*n* = 2), clinical-development, real-world, and causal methodology (*n* = 6), and reporting, safety, and ethics (*n* = 8). Domain assignments were non-exclusive because individual references could inform more than one predefined question; the complete reference-level mapping is provided in Supplementary Table S2. Throughout the review, framework-level proposals are distinguished from empirically demonstrated findings. Framework-level elements define testable development logic and evidence requirements, whereas empirical statements are limited to observations directly supported by the cited source-specific literature. Table 1 summarizes the predefined review questions and corresponding translational outputs.

**Artificial Intelligence and Open Scientific Software Assistance:** Manuscript preparation was supported by freely accessible generative AI models—GPT-5.6 Luna (OpenAI, San Francisco, CA, USA), Claude Sonnet 5.5 (Anthropic, San Francisco, CA, USA), and Gemini 3.8 Flash (Google DeepMind, Mountain View, CA, USA)—used solely for editorial organization, language refinement, structural outlining, and the development of schematic figures depicting membrane microdomains, cellular interfaces, and translational workflows. Literature mapping, evidence retrieval, and citation-context analysis were performed with open biomedical infrastructure: Semantic Scholar (Allen Institute for AI, Seattle, WA, USA), Europe PMC (EMBL-EBI/NIH, Hinxton, UK/Bethesda, MD, USA), and OpenAlex (OurResearch, open scholarly knowledge graph).These tools were employed exclusively for organizational, linguistic, visualization, and literature-navigation tasks; they were not used as sources of scientific evidence or to generate, analyze, or interpret primary experimental data. All outputs were critically reviewed, cross-verified against primary literature and source databases, and approved by the authors, who retain full responsibility for the integrity and accuracy of the manuscript.

## 3. The Treatment-Option Gap

### 3.1. A Dynamic Operational State for Prospective Research

The term treatment-exhausted is used here only as an operational research construct. It describes a time-dependent state in which a patient with active advanced cancer has no immediately suitable, accessible, evidence-based disease-directed option that is acceptable to the patient and treating team. The state may arise from progression, cumulative toxicity, organ dysfunction, impaired Eastern Cooperative Oncology Group (ECOG) performance status, comorbidity, prior intolerance, trial exclusion, geography, financial or logistical barriers, or informed patient preference [12,13]. It is not synonymous with terminal illness, hospice eligibility, futility, or the absence of every conceivable standard treatment. Table 2 distinguishes this state from treatment ineligibility, intolerance, trial ineligibility, and preference-constrained care.

The classification must be reassessed whenever the clinical situation changes. Infection, obstruction, anemia, pain crisis, medication effects, malnutrition, or reversible organ dysfunction may transiently worsen performance status and should be addressed before treatment ineligibility is assigned. Conversely, a patient with preserved performance status may lack a suitable option because biomarker-defined therapies and relevant trials have been depleted. This distinction matters because performance status is prognostic, treatment-modifying, and subject to interobserver variation [14].

### 3.2. Proportionality Requires Better Evidence, Not Lower Standards

Exclusion of patients with poor performance status or comorbidity preserves internal validity but limits generalizability; indiscriminate inclusion can expose participants to disproportionate harm. Contemporary recommendations therefore support broader eligibility while retaining clinically justified safety exclusions and stratifying analyses by performance status and comorbidity. For emerging host-directed products, proportionality should reduce avoidable procedural burden while preserving manufacturing quality, ethics review, informed consent, adverse-event capture, complete denominators, and auditable data. A low-volume intradermal procedure may be less burdensome than an infusion and should be paired with source-appropriate control of biological, infectious, immunological, and manufacturing risks [15]. Figure 1 summarizes the resulting treatment-option gap and the proportional translational response.

## 4. Source-Aware Taxonomy and Product Identity

### 4.1. Source Architecture and Recovered Product Class Are Orthogonal Descriptors

A biological source describes where a preparation begins; a product class describes what is recovered after processing. These dimensions are related but not interchangeable [16].

Human immune cell-conditioned systems may yield soluble mediators, extracellular vesicle-enriched fractions, membrane fragments, non-vesicular lipid–protein assemblies, or mixed extracellular products. Other human cell types may yield structurally similar but functionally distinct materials. Non-human, plant, microbial, manufactured phytochemical, recombinant, and synthetic systems introduce different composition, residual, immunogenicity, adventitious-agent, and manufacturing questions. Mechanism, clinical indication, and regulatory status should therefore be established independently for each source architecture. Microbial systems can release protein-containing membrane vesicles with source-specific cargo and biosafety implications [17], whereas controlled recombinant or synthetic lipid–protein assemblies can be engineered with defined size and composition.

The source-aware approach is essential because the same compositional label can conceal materially different risk and evidence profiles. Equivalence between a matrix derived from human dendritic cell conditioning and materials from other source architectures therefore requires direct analytical and functional evidence. Conversely, different sources may converge on some analytical attributes without sharing functional potency or clinical behavior.

Source, culture or extraction conditions, recovery process, composition, active attributes, intended use, route, and claims must therefore be documented independently [18]. Table 3 maps each source architecture to its minimum evidence burden.

### 4.2. Cell-Free as a Verifiable Manufacturing Attribute

Cell-free means that viable source cells are not intended to be the administered active material. It does not specify whether the preparation contains extracellular vesicles, soluble proteins, lipoprotein particles, membrane fragments, nucleic acids, metabolites, or mixed assemblies. These components may differ in biodistribution, stability, potency, and risk. A cell-free label should therefore be accompanied by an operational description of cell removal, clarification, concentration, fractionation, enrichment or depletion, and the analytical methods used to support the final nomenclature.

Extracellular vesicle terminology should follow the Minimal Information for Studies of Extracellular Vesicles 2023 (MISEV2023) principles. A preparation should not be called an exosome solely because particles fall within a particular size range or express a small marker panel. An extracellular vesicle-enriched product requires orthogonal evidence addressing particle morphology or topology, abundance, size distribution, characteristic and non-vesicular proteins, contaminants, and source-cell context. Conversely, a non-vesicular claim requires a defined operational depletion threshold and evidence that relevant vesicular structures are below that threshold [19]. Figure 2 separates these recovered product classes within the immune cell-derived branch.

### 4.3. Conditional Use of “Phospholipoproteomic Matrix” as a Composition-Led Descriptor

The expression “phospholipoproteomic matrix” may be used conditionally for a recoverable extracellular lipid–protein material in which phospholipid-containing structures and proteinaceous components form reproducible assemblies that contribute to measured biological behavior. It is particularly useful for complex preparations that are neither conventional live-cell products nor purified single molecules and whose relevant activity depends on an integrated architecture. Product-specific use should be supported by measured composition, orthogonal structural evidence, activity-linked attributes, and source-appropriate quality controls [20].

Analytical qualification should demonstrate association or co-organization rather than simple co-presence of lipids and proteins. Depending on the product, the evidence package may combine orthogonal particle analysis, lipidomics, proteomics, density or size fractionation, co-elution, cross-linking or interaction assays, microscopy, detergent or solvent perturbation, and activity-guided depletion or add-back experiments [21]. A manufactured phytochemical–phospholipid complex therefore requires demonstrated protein integration before receiving a phospholipoproteomic descriptor, while a secretome-derived preparation requires evidence that its lipid–protein architecture is reproducible and functionally relevant [22].

Published developmental studies of immune cell-conditioned extracellular lipid–protein preparations now provide a seven-publication evidence lineage spanning platform definition, immune-fingerprint preservation, multistage monitoring, real-time functional profiling, ex vivo traceability, microdomain-oriented mechanistic synthesis, and processed-product proteomic stability. Together, these studies demonstrate that this dendritic cell-conditioned product lineage is experimentally tractable and can be organized into an auditable development sequence beginning with a locked process, quantitative release specifications, and mechanism-linked potency. Table 4 summarizes the recovered product classes and the minimum evidence required to support their respective nomenclature.

## 5. Analytical Characterization and Potency

### 5.1. Minimum Chemistry, Manufacturing, and Control Package

Reliable clinical interpretation depends on consistent lot identity and quantified control of process residuals. For complex extracellular products, characterization must connect source and process history to final composition, purity, microbiological safety, potency, stability, and comparability. The package should distinguish the intended material from process blanks, degraded preparations, unrelated source material, and post-change lots. Protein concentration should therefore be complemented by orthogonal identity and mechanism-linked potency measures. Table 5 consolidates the minimum chemistry, manufacturing, and control package before prospective human evaluation [23].

### 5.2. Potency Must Bridge Product and Biology

Potency is the analytical bridge between manufacturing consistency and a plausible biological function. For complex biological products, an assay should be linked to a defined mechanism or mechanistic family, discriminate active material from process blanks and degraded or irrelevant controls, and preferably be stability-indicating. When several functions are proposed, an assay matrix may combine a primary functional test with supporting biochemical, structural, or phenotypic measures [24].

Candidate assays for immune-conditioned products include controlled dendritic cell maturation, antigen-presentation competence, T-cell activation under standardized stimulation, suppression-reversal models, reporter assays, membrane-order measurements, or source-appropriate cell-interface models [25]. Each assay requires qualified cells or donors, a defined replicate structure, system-suitability controls, analytical precision, acceptance limits, reference material, and a plan established before clinical lots are tested. Release criteria should be prospectively defined before clinical-lot testing and supported by reproducible assay performance.

### 5.3. Potency Must Remain Source- and Claim-Specific

The same assay cannot automatically qualify products from different sources. An immune cell-conditioned material may justify antigen-presentation or immune-synapse readouts [26], whereas a dermal stromal-cell preparation may require structural compatibility or barrier-relevant assays. Plant-derived or phytochemical matrices may require digestion, permeability, metabolic stability, and bioavailability models before immune claims are entertained. Non-human products need source-specific immunogenicity and contaminant assessment. Defined synthetic assemblies may permit narrower mechanism assays but still require proof that the selected assay predicts the intended biological effect.

This source–claim alignment prevents a common translational error: choosing an assay because it produces a favorable signal rather than because it measures the intended product function. The potency strategy should be prespecified from the intended mechanism, clinical role, and route, then challenged by fractionation, depletion, add-back, inactive lots, and dose- or exposure-ranging experiments [27].

### 5.4. Assay Qualification, Variability, and Reproducibility

Assays used to characterize complex cell-free immune-conditioned products should not only be evaluated for biological relevance but also for analytical reproducibility across the sources of variation expected during development. Relevant variability includes within-run and between-run precision, donor-to-donor differences in primary or immune cell systems, lot-to-lot product variability, operator effects, instrument or reagent changes, and, where transferability is intended, inter-laboratory performance. These sources of variation should be characterized prospectively so that biological differences can be distinguished from assay noise [18,23,24,27].

System-suitability controls, predefined reference materials, positive and negative controls, and prespecified acceptance ranges should accompany mechanism-linked potency and functional assays whenever feasible [22,23,24]. Reference standards may include a qualified product lot, an internal biological standard, a defined control material, or a standardized cellular response benchmark appropriate to the intended assay. Donor-dependent systems require particular attention because baseline phenotype, activation state, and intrinsic responsiveness can materially influence measured effects. Experimental designs should therefore define donor-selection criteria, replicate structure, normalization procedures, and rules for handling technically unsuitable biological systems [18,22,24].

Assay qualification establishes fitness for a defined developmental purpose, whereas formal validation requires predefined evidence of performance characteristics such as precision, specificity, robustness, range, and reproducibility appropriate to the intended use [24]. Early exploratory assays may therefore be qualified for mechanism discovery or biomarker selection without meeting the evidentiary standard required for lot release or pivotal clinical interpretation. As development progresses, assays supporting manufacturing decisions, potency specifications, dose selection, or confirmatory biological claims should undergo increasingly stringent qualification and, when appropriate, formal validation [22,23,24]. Transfer across laboratories should additionally assess method comparability, reference-standard performance, analytical drift, and predefined criteria for successful method transfer.

For complex products whose activity may depend on multiple interacting components, no single assay is necessarily sufficient to capture overall biological function. A fit-for-purpose assay strategy may therefore combine a primary mechanism-linked functional assay with supporting structural, biochemical, phenotypic, or kinetic measurements [22,24,27]. The evidentiary value of such an assay matrix depends on reproducibility, prespecified interpretation rules, and demonstrated linkage between the measured response and the intended product function.

## 6. Membrane Microdomains as a Falsifiable Mechanistic Bridge

### 6.1. Structural Organization of Immune Signaling

Membrane microdomains are dynamic, compositionally ordered regions in which cholesterol, sphingolipids, phospholipids, receptors, adaptor proteins, and cytoskeletal elements are spatially coordinated. They regulate lateral mobility, local concentration, interaction probability, and the persistence of signaling components. Their organization can therefore determine whether receptor engagement produces coherent amplification, incomplete activation, or desensitization [28].

This organizational level is especially relevant to antigen-presenting cells. Effective dendritic cell function requires coordinated display of peptide–MHC complexes, costimulatory molecules, pattern-recognition receptors, adhesion proteins, signaling kinases, and cytoskeletal anchoring systems. A cell may retain these components while losing the spatial coherence needed to assemble competent signaling platforms or stable immune synapses. Structural disruption can therefore produce functional failure without deletion of the molecular machinery [29].

Dendritic cells are themselves heterogeneous. Conventional type 1 and type 2 dendritic cells, plasmacytoid dendritic cells, monocyte-derived dendritic cells, inflammatory dendritic cell states, and ex vivo differentiated populations differ in ontogeny, receptor repertoire, antigen-processing capacity, cytokine programs, trafficking, and responsiveness to tumor-derived signals. Consequently, a mechanism or potency assay demonstrated in one dendritic cell population should not automatically be generalized to another. Source-cell phenotype and differentiation state should therefore be treated as critical product and assay variables [25,26].

For extracellular lipid–protein assemblies, membrane organization provides a plausible bridge between composition and immune engagement. Candidate interactions include surface adsorption, receptor-mediated uptake, endocytosis, lipid exchange, membrane fusion, or intracellular processing [29]. Associated proteins may influence trafficking, adhesion, redox state, or signaling. Each candidate mechanism requires direct evidence beyond compositional detection. Product-specific validation can therefore progress through membrane incorporation, microdomain localization and reorganization, receptor clustering, and associated changes in immune competence [30,31].

An integrative structural synthesis has proposed membrane order, receptor clustering, immune-synapse geometry, intracellular organization, and temporal signal integration as a mechanistic family for dendritic cell-mediated antitumor immunity. A separate quantitative proteomic study showed that membrane-associated and extracellular structural proteins remained broadly detectable and reproducible after concentration, cryopreservation, and lyophilization, with short-term compatibility in non-malignant dermal-derived cells [32]. These evidence layers are complementary: one defines a mechanistic hypothesis, and the other shows that relevant compositional features can survive processing. Together, these evidence layers establish compositional and mechanistic tractability, while in vivo membrane interaction and clinical immune restoration constitute subsequent prospective validation layers. Figure 3 depicts the testable transitions from product composition to human biological engagement.

### 6.2. Sequential Evidence Required for a Microdomain Claim

A direct microdomain claim requires a sequential chain. First, independently manufactured lots must demonstrate reproducible lipid and protein composition. Second, interaction with the target-cell membrane should be shown by traceable labeling, quantitative uptake, membrane fractionation, lipid exchange, or orthogonal localization. Third, membrane order or nanoscale organization should be measured using suitable methods such as Laurdan generalized polarization, fluorescence resonance energy transfer, detergent-resistant membrane analysis, imaging of ganglioside GM1 (monosialotetrahexosylganglioside), single-particle tracking, or super-resolution microscopy. Fourth, relevant receptors and signaling proteins should show altered clustering, mobility, colocalization, phosphorylation, or temporal persistence. Finally, perturbation and rescue experiments should test causality [33].

The functional readout must be linked to the structural change. Membrane association without improved signaling competence is insufficient, and nonspecific changes in membrane fluidity may be neutral or harmful. Mechanistically informative assays should connect structural organization to dendritic cell maturation, antigen presentation, costimulatory organization, immune-synapse stability, T-cell priming, cytokine polarization, or resistance to a defined suppressive challenge [34]. Process blanks, compositionally irrelevant lipids, degraded material, inactive lots, and dose-ranging conditions are required to determine specificity.

Microdomain measurements occupy an intermediate evidentiary level that can support mechanistic coherence and potency qualification; tumor activity and clinical benefit are evaluated independently through prospective clinical endpoints. Terms such as microdomain reconstruction, raft repair, or restoration of immune competence should be reserved for direct, reproducible, mechanism-linked evidence and subsequent prospective confirmation in human samples [35].

### 6.3. Microdomain Reconstruction as a Falsifiable Mechanistic Framework: Operational Definition and Claim Boundary

Microdomain reconstruction is defined here as a product-specific, falsifiable mechanistic framework for evaluating restoration, normalization, or deliberate reconfiguration of the spatial organization and functional coupling of membrane microdomains in immune cells. The framework links structural reorganization to directly measurable functions, including receptor clustering, signal integration, antigen-presentation competence, and immune-synapse stability [36]. A product-specific microdomain-reconstruction claim requires a defined evidence chain comprising reproducible active architecture, verified target-membrane interaction, direct microdomain reorganization, causal receptor/signaling or immune-functional consequences, perturbation/rescue evidence, and prospective confirmation in human samples [37].

The term reconstruction carries a higher evidentiary burden than reorganization. Reorganization denotes demonstrated spatial change whose direction relative to a reference state has not been established; normalization denotes movement toward a prespecified healthy or functional reference; reconstruction or repair requires a demonstrated baseline defect, restoration toward that reference, and recovery of a causally linked function. This evidentiary hierarchy prevents compositional observations from being elevated to therapeutic mechanisms and establishes explicit criteria for product-specific mechanistic validation.

## 7. The Intradermal Route as a Minimally Invasive Immune Interface

Skin is an immunologically active organ containing epidermal Langerhans cells, dermal dendritic cell subsets, macrophages, mast cells, lymphocytes, endothelial cells, and stromal elements. Material placed within the dermis can be captured locally and transported through lymphatics to draining nodes, providing a biologically plausible route for immune engagement [38]. The route has been used in vaccination and dendritic cell strategies, although performance depends on the product, device or needle, dose, formulation, anatomical site, operator, and endpoint.

Selection of the intradermal route should be product-specific rather than assumed for the entire product family. Its suitability depends on formulation volume, particle or assembly characteristics, viscosity, stability after reconstitution, local tissue compatibility, expected cellular interaction, lymphatic access, exposure requirements, and the final delivery configuration. Intradermal administration constitutes a product-specific translational route whose performance is established through the final product-device configuration, including delivery fidelity, tolerability, biological engagement, and clinical assessment.

Operational advantages of the intradermal route include low-volume administration, outpatient delivery, reduced dependence on infusion infrastructure, compatibility with repeated administration, and synchronization with prespecified sampling windows [39]. Their product-specific performance is quantified through delivery, feasibility, tolerability, and biological engagement endpoints. Relevant feasibility outcomes include successful administration, depth or bleb confirmation, leakage, procedure time, local reactions, unscheduled care, patient burden, and adherence.

Key route-performance and safety variables include pain, erythema, induration, ulceration, infection, depot depth, leakage, and operator-dependent variability. Systemic immune effects remain possible. Viscosity, particulate content, excipients, reconstitution, needle or microneedle geometry, and local tissue condition may affect delivery [40]. Early cohorts should therefore standardize anatomical sites, operator training, and administration confirmation. The delivery device, delivered material, and final product-device configuration should therefore be characterized independently with respect to regulatory status, mechanism, safety, and clinical performance. Figure 4 presents the intradermal route as an immune interface, and Table 6 defines its minimum prospective administration and feasibility dataset.

## 8. Patient Selection and Ethical Proportionality

### 8.1. Selection Should Define a Researchable Population, Not a Favorable Subset

An initial prospective study should avoid a pan-cancer population defined only by lack of options. Histology, disease tempo, prior therapy, immunogenicity, organ involvement, corticosteroid exposure, and prognosis can overwhelm a small signal-seeking cohort. A defensible design uses one or a small number of clinically coherent strata, documents why standard treatment is unsuitable, and applies stratified stopping rules. Table 7 organizes the eligibility domains for such an initial cohort [41].

Tumor types differ substantially in baseline immunogenicity, antigen-presentation competence, mutational and neoantigen burden, stromal exclusion, myeloid suppression, prior exposure to immune-modifying therapies, corticosteroid requirements, disease kinetics, and imaging behavior. Early studies should therefore favor biologically and clinically coherent disease strata rather than pooled pan-cancer cohorts unless a strong cross-histology biomarker hypothesis is prespecified.

Participants should retain sufficient reserve to complete the initial biological engagement window unless the study is explicitly palliative or feasibility-focused. Excluding patients expected to deteriorate imminently is not intended to manufacture favorable outcomes; it protects participants from burdensome research and reduces informative missingness. Prognosis should be documented, multidisciplinary where feasible, and revisited. Supportive and palliative care must continue irrespective of participation [42].

### 8.2. Consent Should Preserve Evidentiary Clarity and Patient Autonomy

The consent process should state that the intervention is investigational, biological activation does not equal cancer control, the optimal dose and schedule are unknown, and participation may not provide direct benefit. Research procedures should be distinguished from clinical care; alternatives, including supportive care and standard treatment when available, should be explained. Relevant financial and intellectual property interests must be disclosed. Scarcity, urgency, exceptional access, and certainy language should be avoided [43].

Independent eligibility confirmation or consent review is desirable when investigators also hold commercial or intellectual property interests. A proportionate independent safety mechanism should review serious events, unexpected immune phenomena, rapid deterioration, and deaths. Public registration and protocol availability should precede enrollment. The vulnerability of the population strengthens rather than weakens the need for complete denominators and transparent negative outcomes [44].

## 9. NAM-Aligned Nonclinical Evidence Architecture

### 9.1. NAMs Answer Defined Nonclinical Questions

New approach methodologies comprise in vitro, ex vivo, in chemico, computational, organoid, microphysiological, and other human-relevant approaches used to answer defined questions in product development. Their value depends on a stated context of use, transparent study design, system suitability, reproducibility, and evidence that the method predicts or measures the decision-relevant attribute. NAMs provide decision-relevant evidence when applied within a predefined, fit-for-purpose context of use; their regulatory weight depends on method qualification, validation, study quality, and the specific question addressed [45].

For complex cell-free products, a NAM package may integrate analytical fingerprinting, standardized human cell systems, primary-cell or co-culture assays, patient-derived ex vivo materials, real-time label-free kinetic imaging, organotypic skin or immune models, cytokine and immunophenotypic profiling, multi-omics, and computational integration. Such evidence can support identity, lot comparability, mechanism-linked potency, target-cell interaction, hazard identification, biomarker selection, dose or exposure justification, and the decision to proceed to first-in-population evaluation [46,47]. Table 8 maps each NAM-aligned module to its decision question and claim boundary.

Regulatory initiatives increasingly support reduction or replacement of selected animal studies when an evidence-based, fit-for-purpose alternative provides equal or greater decision value. Current U.S. Food and Drug Administration (FDA) draft guidance emphasizes validation frameworks, context of use, study quality, and the role of NAMs in improving the human relevance of nonclinical evidence. A separate oncology draft guidance describes circumstances in which streamlined nonclinical programs and weight-of-evidence approaches may reduce unnecessary animal testing for certain oncology biologics and conjugated products. These initiatives support streamlined, human-relevant nonclinical development, while prospective human evidence remains the basis for clinical claims [48,49].

### 9.2. Published Evidence Lineage

The framework is source-general by design, with its most developed empirical implementation represented by dendritic cell-conditioned extracellular preparations. The seven-publication lineage provides the worked developmental application of the architecture. Transfer to additional source architectures requires independent source-specific analytical and functional confirmation.

The reviewed publication sequence comprises seven studies in which each article addresses a distinct developmental question rather than repeating a single platform claim. The sequence begins with definition and functional characterization of a dendritic-secretome-derived cell-free lipid–protein preparation, adds preservation of immune-associated fingerprints across processing conditions [50], and then extends through quality and immune-monitoring architecture, label-free kinetic profiling, ex vivo traceability, microdomain-oriented mechanistic synthesis, and processed-product proteomic stability.

This lineage is valuable because it connects product composition to increasingly decision-relevant evidence while keeping analytical identity, functional potency, mechanistic interpretation, and prospective clinical outcomes as separate layers. It supports the feasibility of a staged development program and directly informs the minimum evidence package, Module A biological engagement strategy, and Stage 0–4 validation roadmap proposed in this review. Table 9 maps the seven publications to their principal contribution and translational role.

### 9.3. Positioning NAMs Within the Evidence Sequence

NAMs can reduce uncertainty before human exposure and, when sufficiently validated, can replace selected animal studies for defined contexts of use. Their strongest role in this framework is to qualify identity, comparability, mechanism-linked potency, target-cell interaction, hazard signals, and biomarker selection before entry into prospective human evaluation [51]. Patient-level and tumor-level claims then progress through prespecified human evidence layers addressing tolerability, dose, biological engagement, function, tumor activity, and comparative benefit.

For terminological clarity, “Module A”, “Module B”, and “Module C” denote proposed clinical assessment windows, while “Stage 0” through “Stage 4” denote evidence progression from analytical readiness to comparative validation. NAM-aligned evidence is concentrated in Stages 0 and 1 and supports entry into Stage 2 when product quality, mechanism-linked assays, route performance, and safety monitoring are sufficiently defined.

## 10. A Three-Module Translational Protocol Architecture

The architecture separates early administration feasibility, safety, and biological engagement from later patient-centered and tumor-level outcomes. Modules A–C define analytical and temporal assessment windows that organize an evidence-building sequence from administration fidelity and early biological engagement to functional consolidation, metabolic activity, structural response, and durability. Dose, frequency, duration, and progression between modules are determined by product characterization, mechanism-linked potency, nonclinical safety, stability, and prospectively collected first-in-population data. Disease-specific urgency may require earlier imaging or treatment modification, and clinical necessity takes precedence over research scheduling. Figure 5 shows the temporal logic; Table 10 states module-specific objectives, measurements, and progression criteria [52]. These domains are ordered for measurement and evidence development; they do not represent validated surrogate steps in a causal sequence, and any proposed surrogate relationship requires independent validation.

The architecture improves interpretability by prespecifying when each claim can be assessed and by supporting explicit continue, adapt, or stop decisions. Each module retains independent evidentiary weight while contributing to the integrated longitudinal interpretation.

### 10.1. Module A: Safety and Biological Engagement

Module A spans baseline through approximately day 28 and asks whether the product can be administered reproducibly with acceptable acute risk and whether it generates a prespecified biological signal. Safety events should be graded using the National Cancer Institute Common Terminology Criteria for Adverse Events (CTCAE), version 6.0 [53], and should include local reactions, systemic symptoms, laboratory changes, immune-mediated events, serious adverse events, early deaths, and all-cause discontinuation. Denominators should be reported per participant and per administration, with complete accounting for participants who receive no dose or discontinue early.

Biological engagement should use a compact, qualified panel selected from the proposed mechanism [54]. Candidate variables include CD69 and CD25 within defined CD4 and CD8 parent populations, monocyte HLA-DR, interferon-gamma, interleukin-10, and a functional responsiveness assay. Sampling time, processing delay, anticoagulant, storage, assay batch, gating strategy, limits of detection, and repeatability must be controlled. A directionally favorable biomarker change remains exploratory unless the threshold and multiplicity plan were prespecified.

### 10.2. Module B: Functional Consolidation

Module B extends approximately from days 29 to 84 and asks whether any biological signal is accompanied by a sustained patient-level consequence. The minimum set should include the Eastern Cooperative Oncology Group (ECOG) or Karnofsky Performance Status (KPS), the European Organisation for Research and Treatment of Cancer Quality of Life Questionnaire Core 30 (EORTC QLQ-C30), selected Patient-Reported Outcomes version of the Common Terminology Criteria for Adverse Events (PRO-CTCAE) item [55], analgesic and corticosteroid use, weight, hospitalization, and ability to continue planned care. All supportive-care changes and clinically important cointerventions must be time-stamped.

A functional endpoint should be defined as change from baseline sustained across more than one assessment or as time to definitive deterioration [56]. Isolated clinician-rated improvement is insufficient. Death, hospitalization, treatment discontinuation, and inability to complete questionnaires should be incorporated into the estimand rather than removed as missing observations.

### 10.3. Module C: Metabolic, Structural, and Durability Assessment

Module C evaluates tumor-level activity and durability, generally from approximately day 84 onward. Structural imaging should use Response Evaluation Criteria in Solid Tumors version 1.1 (RECIST 1.1) or an appropriate disease-specific standard; immune-adapted confirmation may use immune RECIST (iRECIST) when indicated. Metabolic imaging should follow a standardized 18F-fluorodeoxyglucose positron emission tomography/computed tomography (18F-FDG PET/CT) protocol using European Organisation for Research and Treatment of Cancer (EORTC) criteria, PET Response Criteria in Solid Tumors (PERCIST), or a prespecified immune-adapted framework. Anatomical and metabolic criteria must be reported separately; iRECIST is not a PET criterion.

New or increased 18F-FDG uptake can reflect malignant progression, immune cell infiltration, infection, granulomatous inflammation, recent procedures, marrow stimulation, or technical inconsistency. Apparent pseudoprogression should remain unconfirmed or indeterminate until longitudinal evidence supports the interpretation. Clinically threatening deterioration should not be delayed for confirmatory imaging, and blinded central review is strongly preferred in small cohorts [57,58].

## 11. Integrated Monitoring Across Distinct Endpoints

An integrated framework is useful only when the meanings of individual domains are preserved. Biological engagement, pharmacodynamic change, immune response, patient-centered outcomes, tumor-level activity, and survival should therefore be analyzed as distinct evidentiary domains rather than interchangeable surrogates.

Product quality, safety, immune engagement, function, metabolism, structure, and clinical benefit require separate denominators, windows, analysis rules, and uncertainty. Integration should occur through prespecified longitudinal hypotheses, not through an unvalidated composite that permits a favorable domain to cancel deterioration elsewhere [59]. Table 11 defines the core outcome set, interpretive threats, and minimum reporting for each domain.

A coherent trajectory may include early biological engagement, stable or improved function, later metabolic reduction, and confirmed structural control. This pattern is stronger than an isolated signal but remains associational without a comparator. Discordance is equally informative. Engagement without function or tumor control may indicate an irrelevant mechanism, inadequate exposure, or resistant disease. Functional improvement without tumor response may represent supportive benefit or confounding. Metabolic improvement without structural confirmation requires technical review and follow-up.

## 12. Falsifiable Predictions and the Claim-to-Evidence Ladder

A translational framework gains scientific value when it generates falsifiable predictions. The following predictions define prospective tests of mechanistic coherence and clinical relevance. Temporal ordering alone does not establish causality. Biological engagement followed by functional improvement or tumor-level activity may be compatible with the proposed mechanism but does not establish that the earlier event caused the later one. Causal interpretation requires convergent evidence from mechanistic perturbation, dose- or exposure-response relationships, appropriate negative or inactive controls where feasible, replication, and—where sample size, temporal structure, and causal assumptions permit—exploratory mediation analyses, followed ultimately by comparative evidence. In small early-phase cohorts, mediation analyses should be regarded as exploratory and should not be interpreted as independent proof of causality.

- A reproducible biological engagement signal should occur within a prespecified window and should be absent or attenuated with process blanks, inactive lots, or mechanistically irrelevant controls.
- The magnitude of engagement should show an exposure-, dose-, or potency-related relationship before mechanistic relevance is claimed.
- A proposed microdomain mechanism should be weakened when membrane interaction, structural reorganization, or perturbation/rescue evidence is absent.
- Participants without early engagement would be expected to have a lower probability of subsequent functional or tumor-level benefit; failure of this association would weaken the proposed chain.
- Functional improvement should remain after adjustment for corticosteroid changes, analgesia, transfusion, infection treatment, radiotherapy, and other time-varying cointerventions.
- Metabolic findings should be reproducible under standardized acquisition and should not depend on excluding progression, early death, or missing follow-up scans.
- Repeated administration requires lot-level identity and potency within prespecified comparability limits; outcome heterogeneity associated with lot drift should trigger investigation or suspension [60].
- A favorable single-arm signal should attenuate under concurrent comparison or target-trial emulation if it is primarily due to selection, regression to the mean, or delayed effects of prior therapy [61].

## 13. Prospective Development Pathway

An initial first-in-population study is best positioned to establish analytical and operational readiness, prospective feasibility, acute safety, and biological engagement, with tumor response captured as a prespecified exploratory outcome. This sequence creates a stronger evidentiary foundation for subsequent evaluation of disease-control and clinical-benefit claims [62]. Figure 6 formalizes the claim-to-evidence ladder and the evidence required at each higher level.

Stage 0 locks the manufacturing process, release tests, potency strategy, stability, source-specific controls, and comparability plan. Stage 1 qualifies mechanism-linked assays and the intended administration procedure. NAM-aligned evidence is concentrated in these stages and may support the decision to enter Stage 2. Stage 2 is a small, prospectively registered first-in-population cohort with conservative stopping rules and exposure-denominated safety [63]. Stage 3 tests a predefined signal in a clinically coherent cohort with repeated biomarkers, validated patient-reported outcomes, and blinded imaging. Stage 4 uses a concurrent comparator, randomized design, or carefully specified target-trial emulation if randomization is infeasible. Figure 7 summarizes this contingent development pathway.

Advancement requires that each evidence layer answer its own question. Unresolved uncertainty should be carried forward explicitly rather than declared resolved by a favorable observation in another domain. NAM evidence should not be used to move directly from analytical readiness to comparative clinical claims.

### 13.1. A Pragmatic First Prospective Study

The initial study is structured as a prospective, single-arm, signal-seeking feasibility study rather than an efficacy trial. It enrolls a narrowly defined advanced-solid-tumor stratum with documented treatment ineligibility, uses one locked manufacturing process and formulation, justifies dose exploration from nonclinical evidence, and defines day-28 biological engagement as the principal mechanistic endpoint. Safety, administration fidelity, function, and complete follow-up would be co-primary or key secondary domains; imaging would be exploratory and centrally reviewed.

Every enrolled participant should be presented, including those who receive no dose, discontinue, die, or lack follow-up imaging. Estimation-focused or Bayesian methods may be more informative than binary null-hypothesis testing in small cohorts [64]. Progression thresholds should consider effect magnitude, uncertainty, safety, and data completeness. A high response estimate among a small subset of evaluable survivors is insufficient.

### 13.2. Comparative Validation and Real-World Evidence

After feasibility, product consistency, safety, and biological engagement are established, comparative validation should address the intended clinical role. The comparator may be optimized supportive care, best supportive care, standard therapy under equipoise, or a delayed-start design. The primary endpoint should match the claim: function or symptom burden for supportive use; confirmed tumor response or progression for antitumor activity; and survival or quality-adjusted survival for clinical benefit.

Real-world data can supplement but not automatically replace a comparator. A target-trial emulation requires explicit eligibility, treatment strategies, assignment procedure, time zero, follow-up, outcomes, causal contrast, and analysis plan, and should follow the TARGET reporting guideline. Historical controls are vulnerable to changes in imaging, supportive care, molecular testing, subsequent therapy, and referral patterns. Real-world evidence is most useful when data provenance, treatment assignment, missingness, and temporal alignment are transparent [65].

## 14. Safety, Governance, Reporting, and Claim Discipline

Complex biological materials require safety surveillance beyond common injection-site reactions. Potential risks include microbial or endotoxin contamination, unintended inflammatory activity, hypersensitivity, immune dysregulation, thrombosis, organ-specific inflammation, interactions with anticancer therapy, and residual source- or process-derived material. The safety plan should be based on the actual source, composition, route, dose, patient comorbidity, and nonclinical findings rather than on an assumption that “cell-free” means inherently safe [66].

Every human protocol should include independent ethics review, prospective registration, a risk-proportionate data-monitoring mechanism, protocol-defined attribution, rapid serious-event reporting, and follow-up after discontinuation. Interventional studies should follow the Standard Protocol Items: Recommendations for Interventional Trials (SPIRIT) 2025 and Consolidated Standards of Reporting Trials (CONSORT) 2025 as applicable; observational evidence should follow the Strengthening the Reporting of Observational Studies in Epidemiology (STROBE) and Reporting of Studies Conducted Using Observational Routinely Collected Health Data (RECORD) guidelines; CTCAE and the Declaration of Helsinki remain relevant to adverse-event capture and ethical conduct. Patient flow, exact denominators, missingness, and reasons for non-evaluability must be explicit [44,52,53,67].

Investigators with ownership, intellectual property, or financial interests should disclose them in protocols, consent materials, registries, manuscripts, and presentations. Independent imaging review, statistical analysis, and safety oversight reduce but do not eliminate conflict-related bias. Negative and inconclusive results should be reported. Source-aware terminology also limits promotional drift by preventing a compositional descriptor from being converted into an unearned therapeutic class. Table 12 states the evidence required for progressively stronger claims [68].

## 15. Strengths, Evidence Boundaries, and Validation Priorities

A principal strength of this review is its integration of product taxonomy, analytical readiness, membrane organization, intradermal feasibility, NAM contexts of use, clinical monitoring, and evidence governance within one auditable architecture. Its source-aware design improves transferability across diverse cell-free preparations while preserving the distinct quality and safety questions created by each origin and manufacturing process [69].

The principal validation priorities are direct linkage of peripheral biomarkers to tumor-relevant immune function, product-specific confirmation of target-membrane interaction and microdomain reorganization, and prospective demonstration that analytical and ex vivo potency measures predict human biological engagement. Proteomic stability and short-term cellular compatibility provide useful intermediate evidence and should be extended through exposure-relevant safety and functional studies [32].

NAM acceptance remains context-of-use dependent [70,71]. Fit-for-purpose qualification should therefore be pursued separately for identity, potency, irritation, systemic hazard, mechanistic prediction, and clinical biomarker selection. The cited FDA documents are draft guidance and are best used to structure validation plans, study quality, and weight-of-evidence decisions while prospective human evidence addresses patient-level benefit.

The review intentionally limits its clinical inferences to published evidence and defines the information required for subsequent product-specific studies: locked manufacturing, complete denominators, standardized intradermal administration, prespecified biological engagement, validated functional outcomes, centrally reviewed imaging, transparent cointerventions, and independent replication. These priorities convert current uncertainty into a practical research agenda for clinically coherent prospective cohorts.

### Clinical Implications of the Framework

The framework informs clinical-development decisions rather than immediate treatment selection. Candidate populations should be defined by a documented treatment-option gap, clinical stability, biological rationale, and capacity to complete an interpretable early assessment. Endpoint selection should follow the evidence layer being tested: administration fidelity and acute safety for route feasibility; prespecified immune and functional measures for biological engagement; validated patient-reported and performance outcomes for patient-level consequences; and standardized metabolic and structural imaging for tumor-level activity.

Safety monitoring should be source-, product-, route-, and exposure-specific. Progression from an initial feasibility cohort to signal-seeking and comparative studies should depend on predefined evidence thresholds rather than isolated favorable observations. The framework thereby guides patient selection, endpoint hierarchy, monitoring intensity, stopping rules, and progression from mechanistic plausibility to clinically testable claims.

## 16. Conclusions

Cell-free immune-conditioned products constitute an emerging immunobiological product family whose development benefits from a vocabulary and evidence architecture capable of preserving biological complexity while maintaining analytical clarity and clinical interpretability. Distinguishing biological source, recovered material, and final product class allows human immune cell-conditioned, other human-cell, non-human, plant-derived, microbial, phytochemical, recombinant, and synthetic systems to be evaluated on their own composition, risk, potency, and intended use.

Extracellular lipid–protein matrix provides a neutral family term, while the phospholipoproteomic matrix becomes appropriate when orthogonal methods demonstrate reproducible, activity-linked lipid–protein architecture. Microdomain reconstruction is defined as a falsifiable mechanistic framework with explicit product-specific evidentiary requirements: reproducible product architecture, verified target-membrane interaction, direct structural reorganization, causally linked immune-function recovery, perturbation/rescue evidence, and confirmation in human samples.

By integrating source-aware taxonomy, analytical qualification, mechanism-linked potency, human-relevant nonclinical evidence, intradermal feasibility, biological engagement, patient-centered assessment, and comparative validation, this review establishes a structured translational architecture for complex cell-free immune-conditioned products. The seven-publication evidence lineage demonstrates the experimental tractability of key components of this development sequence, while the claim-to-evidence framework defines the evidentiary requirements for progressive product-specific validation. Together, these elements provide a disciplined and actionable pathway from analytical qualification through mechanistic and biological assessment to clinically meaningful outcomes and comparative validation.

## Figures and Tables

**Figure 1:**
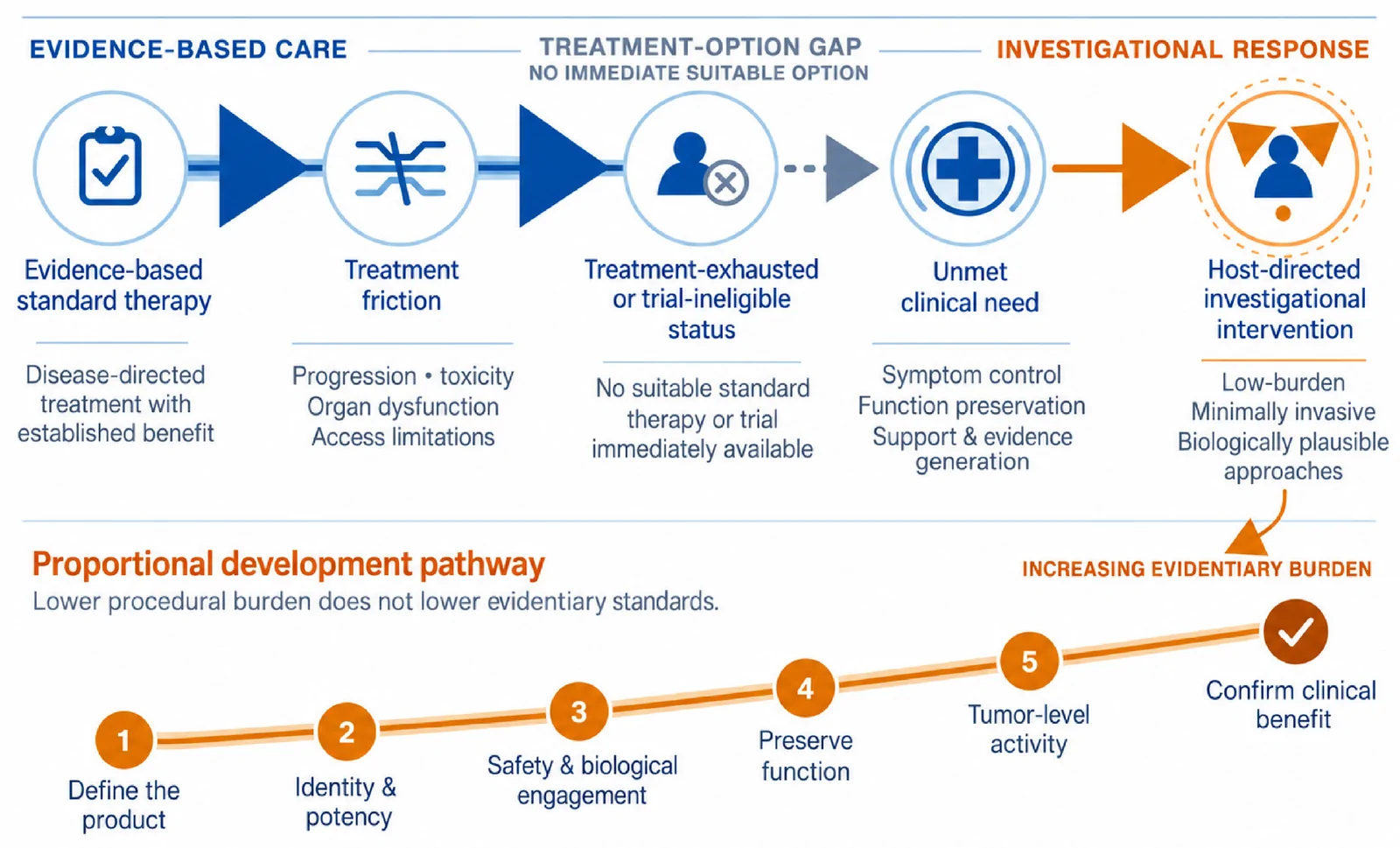
The treatment-option gap and a proportional translational response. The diagram presents conceptual development logic; regulatory pathway, clinical indication, and evidentiary status remain product-specific determinations.

**Figure 2:**
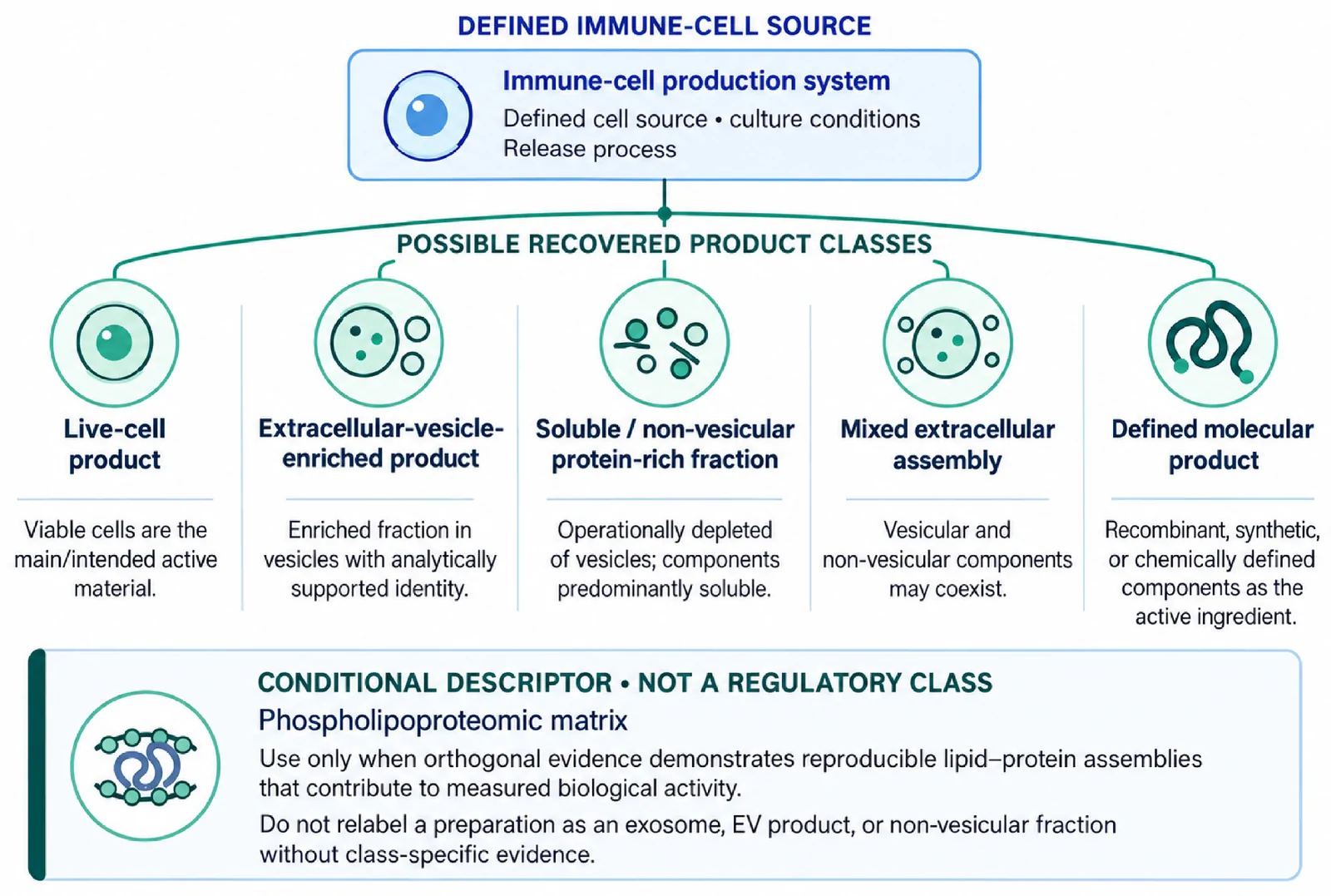
Taxonomy within the immune cell-derived branch of therapeutic materials. “Phospholipoproteomic matrix” is a conditional analytical descriptor within the broader extracellular lipid–protein matrix family and should be used only when lipid–protein assemblies are analytically demonstrated. Source architecture and recovered product class must be reported separately.

**Figure 3:**
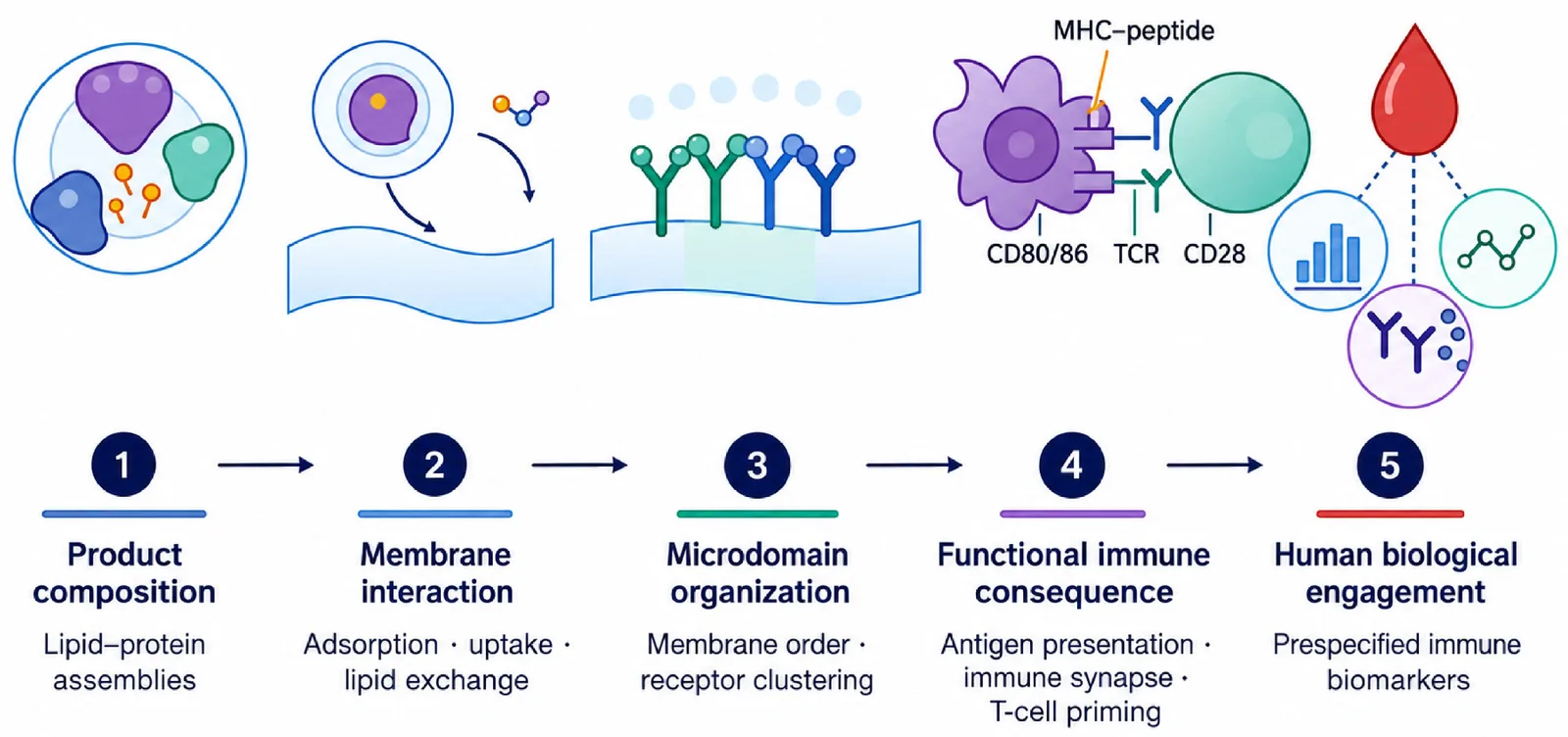
Microdomain reconstruction as a testable mechanistic bridge between product composition and human biological engagement. Arrows indicate the directional evidentiary sequence from product composition to target-membrane interaction, microdomain organization, functional immune consequence, and human biological engagement. Schematic cellular and molecular symbols illustrate representative interactions and readouts and do not represent quantitative data. Each transition requires direct experimental support. MHC, major histocompatibility complex; TCR, T-cell receptor; CD80/86, cluster of differentiation 80 and 86; CD28, cluster of differentiation 28.

**Figure 4:**
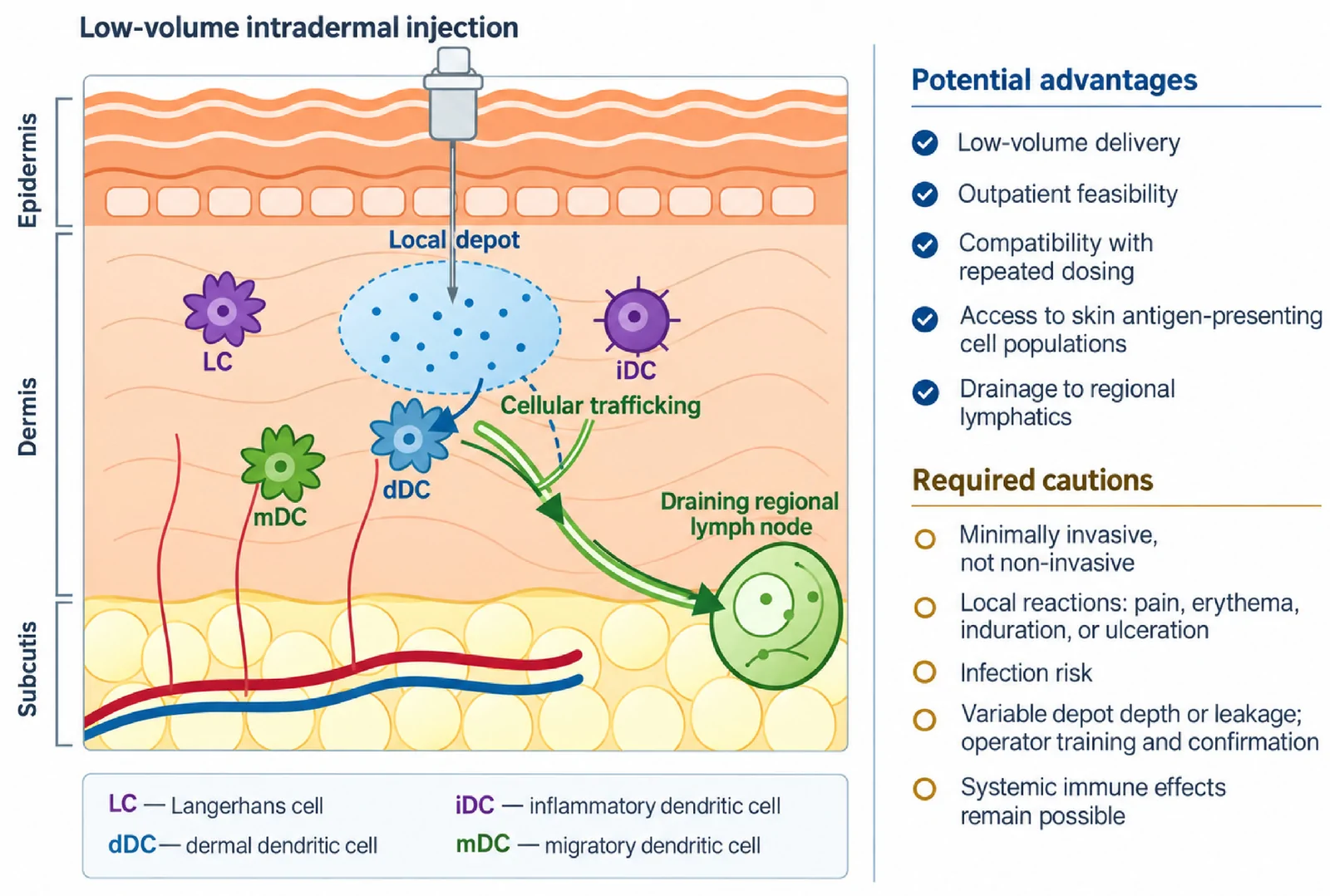
The intradermal route as a minimally invasive immune interface. The diagram distinguishes anatomical and immunological plausibility from product-specific delivery performance, tolerability, and clinical evaluation.

**Figure 5:**
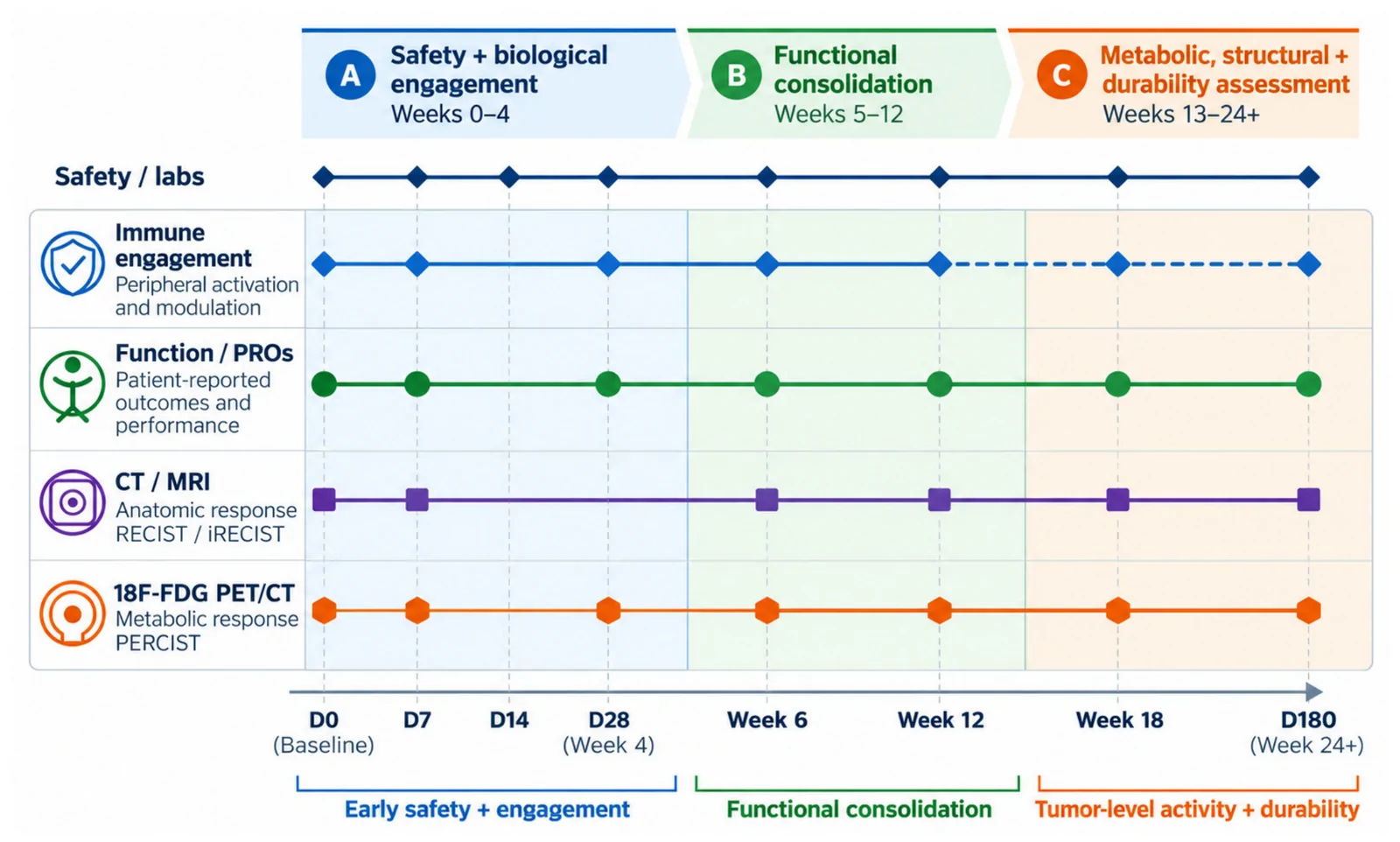
Hypothesis-driven three-module translational protocol. Time points are illustrative research windows rather than a clinical dosing recommendation; disease-specific imaging and safety requirements take precedence. Abbreviations: PROs, patient-reported outcomes; CT, computed tomography; MRI, magnetic resonance imaging; RECIST, Response Evaluation Criteria in Solid Tumours; iRECIST, immune Response Evaluation Criteria in Solid Tumours; 18F-FDG, 18F-fluorodeoxyglucose; PET/CT, positron emission tomography/computed tomography; PERCIST, PET Response Criteria in Solid Tumours.

**Figure 6:**
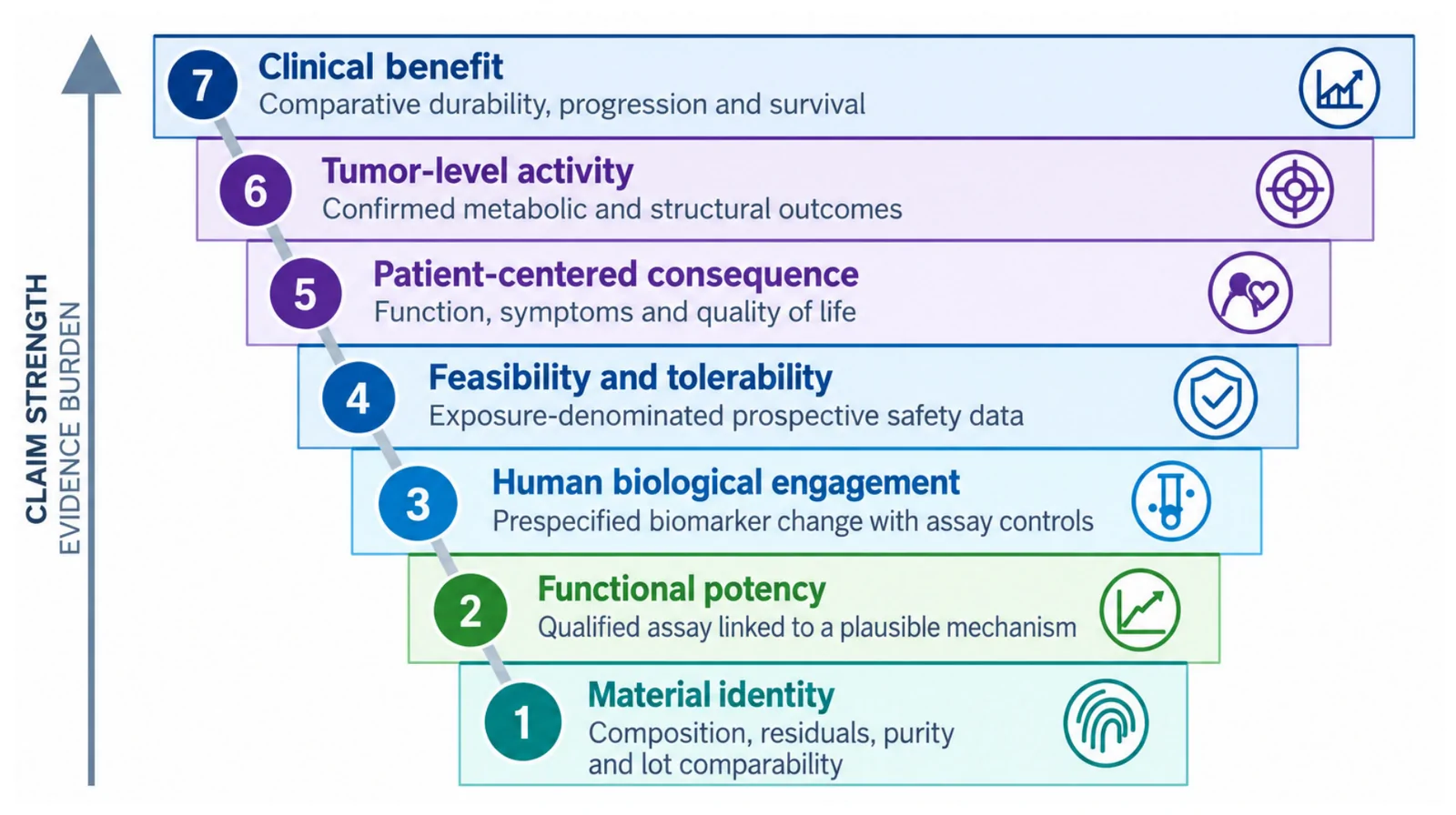
Claim-to-evidence ladder for an emerging cell-free immunobiological platform. Each higher claim requires independent evidence; analytical characterization and biological engagement cannot substitute for clinical validation.

**Figure 7:**
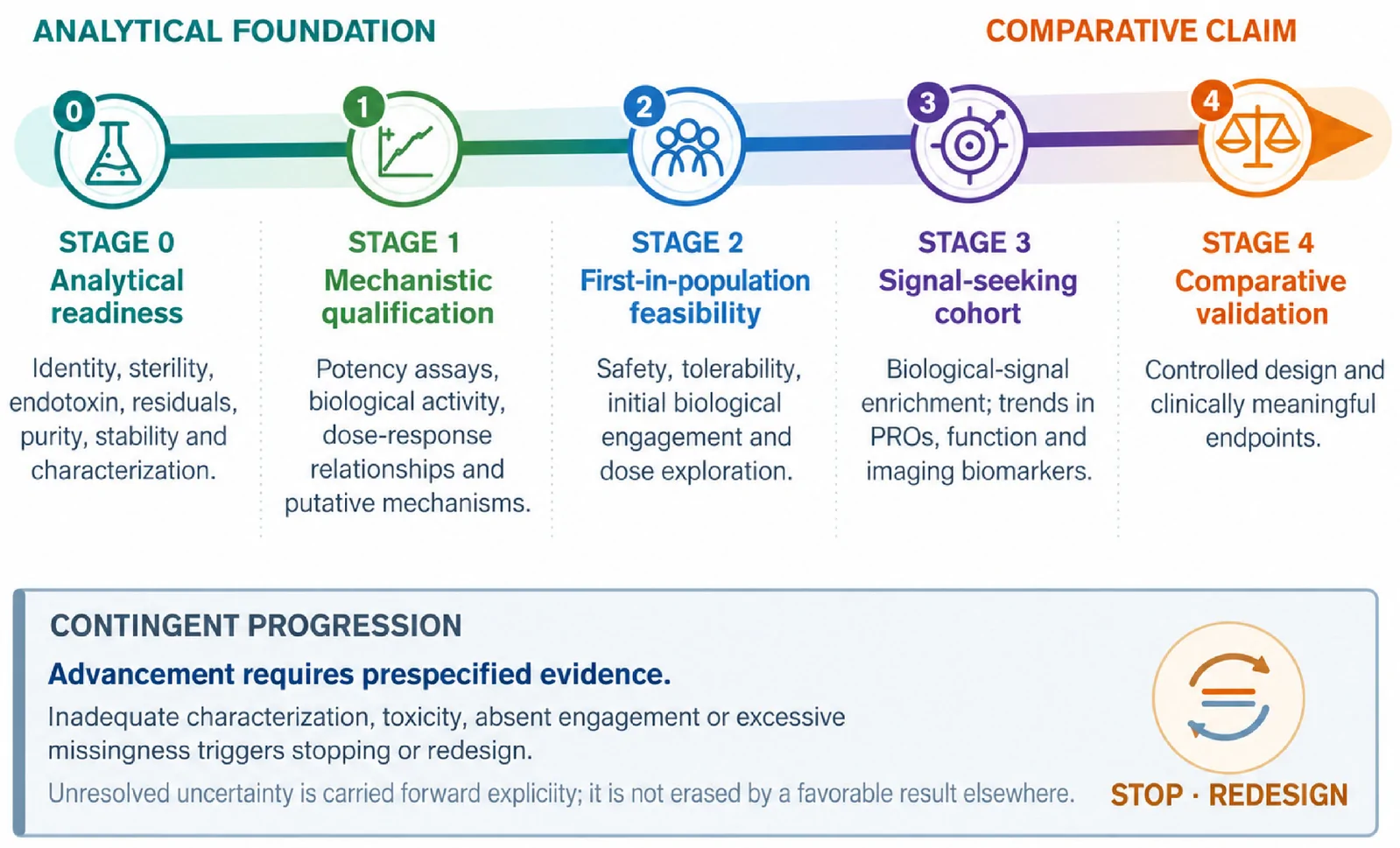
Prospective validation roadmap. Advancement is contingent on prespecified evidence, source- and mechanism-appropriate tests, and explicit stop or redesign rules.

**Table 1:** Predefined review questions and corresponding translational outputs.

Review Domain	Predefined Question	Translational Output
Clinical context	Who can be studied when no immediately suitable standard option or trial is available?	Operational categories, reversible exclusions, reassessment, and protected supportive care.
Source and product identity	What is the biological origin, and what material is actually recovered?	Source-aware taxonomy separated from composition-led product naming.
Analytical readiness	What must be established before biological or clinical interpretation?	Identity, purity, residuals, potency, stability, and lot comparability.
Mechanism	Can lipid–protein architecture alter membrane organization in a measurable and causal manner?	Sequential microdomain evidence chain and mechanism-linked potency.
Route and clinical modules	Why use the intradermal route, and how should outcomes be sequenced?	Prospective administration dataset and three-module immune–functional–tumor assessment.
NAM and evidence governance	What can nonclinical human-relevant methods establish, and where do they stop?	Context-of-use mapping, stop/adapt rules, and claim-to-evidence ladder.

**Table 2:** Operational categories within the treatment-option gap and their research implications.

Category	Operational Definition	Required Checks Before Classification	Research Implication
Treatment-exhausted	No immediately suitable and accessible evidence-based disease-directed option after review of diagnosis, biomarkers, prior therapy, and current standards.	Pathology, molecular testing when relevant, reversible complications, and trial availability.	May be considered for prospective low-burden research with explicit uncertainty and continued supportive care.
Treatment-ineligible	A standard option exists, but present organ function, performance status, comorbidity, or safety risk makes its use unacceptable.	Reversible organ dysfunction, infection, interactions, and accuracy of performance assessment.	The clinical rationale and reassessment date must be documented.
Treatment-intolerant	A potentially useful therapy was stopped or cannot be resumed because toxicity outweighs expected benefit.	Adequacy of toxicity management, dose modification, and causality assessment.	The previous toxicity phenotype should shape eligibility and monitoring.
Trial-ineligible	A potentially relevant study exists, but protocol criteria, timing, geography, or logistics prevent enrollment.	Site contact, document completeness, and remediable eligibility items.	Trial exclusion must not be presented as evidence that an unvalidated intervention is effective.
Preference-constrained	The patient declines an available standard option after informed discussion.	Decision support, misinformation, distress, coercion, and understanding of alternatives.	Autonomy is respected, but refusal does not reduce evidentiary or safety standards.

**Table 3:** Source architectures and their minimum evidentiary implications.

Source Architecture	Potential Recovered Material	Priority Evidence Burden	Additional Evidence Required Beyond Origin
Human immune cell-conditioned system	Immune-associated soluble factors, vesicular and non-vesicular lipid–protein assemblies, membrane fragments, or mixed fractions.	Cell identity and phenotype; donor or bank provenance; conditioning schedule; residuals; identity; mechanism-linked potency; lot comparability.	Direct potency, human biological engagement, and clinical outcome evidence.
Other human-cell secretome	Structural, trophic, extracellular-matrix, membrane-associated, and mixed extracellular components.	Cell identity, passage/state, culture conditions, provenance, composition, residuals, and relevant functional assays.	Mechanism-linked immune-function evidence appropriate to the intended claim.
Non-human biological source	Species-specific proteins, glycans, lipids, extracellular particles, and biofluid components.	Species and tissue source; zoonotic/adventitious agents; xenogeneic antigens; allergens; impurities; batch variability.	Human compatibility, immunogenicity, and product-specific comparability evidence.
Native plant-derived extracellular material	Plant lipids, proteins, nucleic acids, metabolites, and vesicle-like fractions.	Botanical identity; tissue and harvest conditions; isolation; pesticides; mycotoxins; composition; storage; batch control.	Source-specific uptake, potency, and human-relevant safety evidence.
Microbial or fermentation-derived system	Secreted proteins, lipids, membrane vesicles, metabolites, or engineered assemblies.	Strain identity; genetic stability; fermentation controls; endotoxin or cell-wall components; residual host material.	Human-relevant potency, contaminant control, and exposure-based safety evidence.
Manufactured phytochemical–phospholipid/ protein matrix	Defined or semi-defined phytoconstituents associated with phospholipids and, where demonstrated, proteinaceous components.	Exact formulation; stoichiometry; interaction chemistry; colloidal behavior; digestion; bioavailability; stability.	Demonstrated protein integration, mechanism-linked activity, and formulation-specific bioavailability.
Defined recombinant or synthetic assembly	Selected lipids, recombinant proteins, peptides, polymers, or synthetic components under controlled ratios.	Molecular identity; stoichiometry; assembly specifications; impurities; degradation; mechanism; stability.	Assembly-specific functional evidence and confirmation of the intended biological consequence.

**Table 4:** Recovered product classes and minimum naming evidence.

Proposed Label	Minimum Evidence	Additional Evidence for Higher-Level Claims
Live-cell immune product	Viability, identity, phenotype, dose, sterility, functional potency, and stability of administered cells.	Prospective persistence, tumor specificity, and clinical outcome evidence.
Extracellular vesicle-enriched product	Orthogonal particle characterization; EV-associated and non-EV markers; particle-to-protein metrics; source and process controls.	Endosomal-origin studies, mechanism-linked potency, and clinical translation.
Soluble/non-vesicular secretome fraction	Validated cell removal; operational particle depletion; soluble-protein and residual profiling.	Quantified residual membrane material and identification of active attributes.
Mixed extracellular assembly	Reproducible particle, lipid, protein, and soluble-component profiles with explicit acknowledgment of mixed composition.	Mechanistic deconvolution and evidence for the contribution of individual components.
Phospholipoproteomic matrix (conditional descriptor)	Evidence of associated lipid–protein architecture, compositional reproducibility, and activity-linked attributes.	Source definition, release specifications, human safety, dose, and clinical validation.
Defined molecular or synthetic product	Molecular identity, purity, stoichiometry, impurities, and validated assembly specifications.	Demonstration of emergent functions if equivalence to a complex biological matrix is proposed.

**Table 5:** Minimum chemistry, manufacturing, and control package before prospective human evaluation.

Domain	Required Elements	Decision Rule
Source and provenance	Source organism or cell type; donor or bank eligibility when applicable; cell state, passage or differentiation; botanical/strain identity; culture or extraction conditions; chain of custody.	Untraceable or variably defined source material precludes release.
Process definition	Clarification, cell removal, concentration, fractionation, filtration, purification, nuclease steps, excipients, lyo-protection, lyophilization, reconstitution, and container closure.	Critical process parameters must be locked before outcomes are pooled.
Identity and architecture	Protein and lipid profiles; particle or assembly characterization; prespecified identity panel; proteomic/lipidomic fingerprint; architecture-specific orthogonal method.	Identity criteria must distinguish product, blank, degraded material, and unrelated lots.
Purity and residuals	Viable cells, host–cell DNA/RNA, medium proteins, cytokine carryover, reagents, aggregates, particulates, adventitious agents, pesticides, or microbial residues as source-appropriate.	Residual limits and methods must be prespecified and qualified.
Microbiological safety	Sterility or validated rapid method, endotoxin or relevant pyrogen testing, mycoplasma, bioburden, and source-specific contaminants.	Any critical out-of-specification result is a release failure.
Potency	One or more qualified assays linked to a plausible mechanism, with controls, precision, reference material, and acceptance range.	Concentration or an exploratory cytokine readout alone is not potency.
Stability	Real-time and accelerated stability; post-reconstitution hold; freeze–thaw sensitivity; container compatibility; structural and functional stability.	Use is limited to supported storage and handling conditions.
Lot comparability	Predefined similarity ranges across identity, purity, potency, and stability; change-control strategy.	Post-change lots require comparability before clinical data are combined.

**Table 6:** Minimum prospective intradermal administration and feasibility dataset.

Domain	Minimum Variables
Product at administration	Lot; concentration; volume; reconstitution time; appearance; temperature; time from preparation to administration; particulate or viscosity observations.
Technique or device	Needle or device; gauge, length, or microprojection characteristics; angle; site; operator training; evidence of dermal delivery; leakage or failed administration.
Local tolerability	Pain score; erythema and induration diameter; edema; ulceration; infection; duration; intervention required; photographic documentation when appropriate.
Systemic tolerability	Vital signs when indicated; fever; chills; hypersensitivity; inflammatory symptoms; CTCAE grade; attribution; seriousness; immune-mediated events.
Feasibility	Successful administration rate; procedure time; missed or delayed doses; unscheduled visits; patient-reported burden; discontinuation reason.
Concomitant context	Steroids; antibiotics; antipyretics; anticoagulants; systemic therapy; radiotherapy; vaccinations; procedures near the assessment window.

Abbreviations: CTCAE, Common Terminology Criteria for Adverse Events.

**Table 7:** Eligibility domains for an initial prospective signal-seeking cohort.

Domain	Suggested Requirement	Reason
Disease confirmation	Pathologically confirmed advanced solid tumor; current staging; measurable or evaluable disease appropriate to planned imaging.	Reduces diagnostic uncertainty and supports interpretable tumor-level endpoints.
Treatment status	Documented review showing no immediately suitable or accepted standard option, or a clear contraindication or intolerance.	Defines the treatment-option gap without implying futility.
Clinical stability	No uncontrolled infection, acute obstruction, major bleeding, unstable cardiopulmonary condition, or urgent organ-threatening progression.	Reduces immediate non-product risk and competing causes of deterioration.
Performance and prognosis	Prespecified ECOG/KPS range; ability to complete the initial assessment; reason for impaired status documented.	Balances inclusion with feasibility and limits survivor bias.
Immune confounders	Document corticosteroid dose, immunosuppressants, autoimmune disease, infection, transfusion, vaccination, and recent systemic treatment.	Essential for interpreting immune biomarkers and toxicity.
Organ function	Thresholds justified by expected product and procedural risks rather than copied automatically from cytotoxic trials.	Supports proportional eligibility while preserving safety.
Consent and support	Decision-making capacity; realistic explanation of uncertainty; caregiver or logistical plan; access to urgent care.	Protects a vulnerable population and supports follow-up.

Abbreviations: ECOG, Eastern Cooperative Oncology Group; KPS, Karnofsky Performance Status.

**Table 8:** NAM-aligned evidence modules, decision questions, and claim boundaries.

Evidence Module	Representative Methods	Decision Question	Next Evidence Layer Required
Analytical identity and comparability	Proteomics, lipidomics, particle/assembly characterization, residual testing, multivariate fingerprints, stability-indicating assays.	Is the intended material reproducibly present, and are lots/process changes comparable?	Mechanism-linked activity, human tolerability, and prospectively assessed clinical outcomes.
Cell-interface and uptake	Traceable labeling, quantitative uptake, membrane fractionation, lipid exchange, confocal or super-resolution imaging.	Does the product interact with the intended cellular compartment?	Causal functional testing and demonstration of beneficial biological consequences.
Mechanistic potency	Microdomain-order assays, receptor clustering, antigen presentation, immune-synapse, reporter, perturbation and rescue studies.	Does a reproducible, product-linked mechanism produce a defined function?	Prospective human tolerability, biological engagement, and tumor-level assessment.
Human-relevant safety	Primary human cells, organoids, microphysiological systems, immune activation panels, cytokine release, dermal irritation or barrier models.	What hazards are plausible under supported exposures, and which monitoring is required?	Exposure-denominated clinical safety and longitudinal follow-up.
Phenotypic and kinetic profiling	Continuous label-free imaging, viability, morphology, secretomic profiling, patient-derived ex vivo systems.	Are response patterns reproducible across cells, batches, and time?	Prospective responder evaluation with clinically defined outcomes.
Computational integration	Mechanistic modeling, multi-omic integration, Bayesian evidence synthesis, exposure modeling, target-trial planning.	Can available data support a transparent decision or reduce uncertainty?	Empirical confirmation in fit-for-purpose analytical, nonclinical, and clinical systems.

**Table 9:** Published evidence lineage and translational contribution of dendritic cell-conditioned extracellular lipid–protein preparations.

Publication	Evidence Layer	Principal Contribution	Role in the Framework
Cancers 2025 [3]	Platform definition	Introduces a dendritic-secretome-derived cell-free lipid–protein preparation and integrates analytical and functional characterization.	Establishes experimental tractability and a source-to-product starting point.
Biomedicines 2025 [50]	Immune-fingerprint preservation	Reports a proteomic profile exceeding 400 proteins with retention of more than 90% of immune-relevant markers after cryoprocessing.	Supports process resilience, identity panels, and comparability planning.
IJMS 2025 [7]	Monitoring architecture	Organizes product quality, flow cytometry, cytokine profiling, and longitudinal immune assessment.	Informs Module A biological engagement and integrated monitoring.
Biology 2025 [46]	Real-time functional profiling	Uses label-free kinetic imaging to resolve non-cytotoxic phenotypic divergence across tumor-cell models.	Provides a NAM-aligned functional-stratification module.
Biomedicines 2025 [47]	Ex vivo traceability	Links formulation exposure, kinetic observation, and audit-ready documentation without clinical exposure.	Supports potency development, provenance, and reproducible decision records.
IJMS 2026 [6]	Mechanistic synthesis	Formalizes membrane microdomain organization, receptor clustering, immune-synapse geometry, and falsifiable repair hypotheses.	Provides the sequential microdomain evidence chain and perturbation/rescue agenda.
Pharmaceutics 2026 [32]	Processed-product stability	Documents reproducible proteomic features after concentration, cryopreservation, and lyophilization with short-term cellular compatibility.	Supports stability, comparability, and mechanism-linked qualification priorities.

**Table 10:** Module-specific objectives, measurements, and progression criteria.

Module	Primary Objective	Core Measurements	Decision to Progress
A: baseline–day 28	Administration feasibility, acute safety, and biological engagement.	Dose/exposure; CTCAE; local reactions; laboratories; prespecified immune panel; all early discontinuations and deaths.	Release criteria met; no unacceptable toxicity; adequate administration fidelity; interpretable engagement data.
B: days 29–84	Persistence of engagement and patient-centered consequence.	Repeated immune panel; ECOG/KPS; QLQ-C30; PRO-CTCAE; medications; hospitalization; cointerventions.	Signal is sustained or functionally coherent; missingness and confounding remain manageable.
C: days 84–180+	Tumor-level activity and durability.	RECIST/iRECIST; PERCIST or prespecified PET framework; duration; progression; survival; treatment changes.	A predefined signal justifies comparative validation; otherwise stop or redesign.

Abbreviations: QLQ-C30, Quality of Life Questionnaire Core 30; PRO-CTCAE, Patient-Reported Outcomes version of the Common Terminology Criteria for Adverse Events; RECIST, Response Evaluation Criteria in Solid Tumours; iRECIST, immune Response Evaluation Criteria in Solid Tumours; PERCIST, PET Response Criteria in Solid Tumours; PET, positron emission tomography.

**Table 11:** Core outcome set, interpretive threats, and minimum reporting.

Domain	Preferred Variables	Major Threats	Minimum Reporting
Product	Identity, purity, potency, stability, lot, and administered dose.	Lot drift; post hoc assays; concentration treated as potency.	Exact lot-level release data, deviations, and change history.
Safety	Participant- and administration-level adverse events, serious adverse events, immune-mediated events, deaths, and discontinuations.	Under-capture; denominator mismatch; attribution bias.	Prospective CTCAE capture, all-cause events, exposure denominators, and adjudication rules.
Immune engagement	Defined cell subsets, activation markers, cytokines, and functional assay.	Infection; steroids; timing; batch effects; bystander activation.	Assay standard operating procedure, raw and normalized units, baseline and repeated values, multiplicity plan.
Function and PROs	ECOG/KPS, QLQ-C30, PRO-CTCAE, analgesics, activity, and hospitalization.	Observer bias; supportive care; informative missingness.	Repeated measures, responder definition, death/deterioration estimand, cointerventions.
Metabolic imaging	Lean-body-mass-normalized peak standardized uptake value (SULpeak), metabolic tumor volume, total lesion glycolysis, new lesions, immune-related findings.	Acquisition variability; inflammation; infection; evaluable-scan selection.	Uptake time, glucose, scanner/reconstruction, criteria, blinded review, full denominator.
Structural imaging	Target/non-target lesions, new lesions, confirmation, and duration.	Nonstandard timing; mixed response; unblinded assessment.	RECIST or disease-specific criteria and separate iRECIST status where applicable.
Clinical benefit	Progression, hospitalization, treatment-free interval, survival, and quality-adjusted outcomes.	Immortal-time and survivor bias; subsequent therapy; loss to follow-up.	Time origin, censoring, competing events, and complete participant flow.

**Table 12:** Evidence requirements and evidence-proportionate wording for progressively stronger claims.

Claim	Evidence Required	Evidence-Proportionate Wording
Source and product identity	Qualified source documentation, analytical panel, process controls, and lot comparability.	Source-defined, analytically qualified, and lot-comparable product.
Integrated lipid–protein architecture	Orthogonal evidence that lipid and protein components form reproducible, activity-linked assemblies.	Reproducible, activity-linked lipid–protein assemblies demonstrated by orthogonal methods.
Biological activity	Qualified potency assay plus human biological engagement endpoint when clinically studied.	Mechanism-linked potency with prospectively confirmed human biological engagement.
Product-specific microdomain-reconstruction claim	Reproducible active architecture; target-membrane interaction; direct microdomain change; causal receptor/signaling and immune function; perturbation/rescue evidence; prospective human confirmation.	Product-associated microdomain reorganization or reconstruction supported by structural, causal, and human evidence.
Minimally invasive delivery	Prospective delivery performance and tolerability for the final product–device configuration.	Prospectively documented low-volume intradermal feasibility and tolerability.
Supportive patient benefit	Validated function or PRO endpoint, cointervention capture, and a relevant comparator.	Validated improvement in function or patient-reported outcomes with controlled cointervention assessment.
Antitumor activity	Confirmed criteria-based tumor outcome with complete denominator and independent review.	Criteria-based tumor activity with complete denominators and independent review.
Clinical efficacy	Comparative evidence for a clinically meaningful endpoint.	Comparative clinical benefit on a prespecified clinically meaningful endpoint.

## Data Availability

The reproducible search blocks, evidence-domain map, and published evidence-lineage map are supplied in Supplementary Materials (Tables S1–S3). The additional literature-charting materials are available from the corresponding author upon reasonable request.

## Supplementary Materials

The following supporting information can be downloaded at https://www.mdpi.com/article/10.3390/medicina62101964/s1: Section S1. Scope and Search Update; Table S1, Reproducible PubMed/MEDLINE search blocks; Section S2. Evidence-Domain Map; Table S2, Evidence map of the 71-reference interpretive corpus; Section S3. Published product-development evidence-lineage map; Table S3, Published evidence lineage of dendritic-cell-conditioned extracellular lipid–protein preparations; Section S4, Update and Reproducibility Procedure.
