# Supplementary material for: Cell-Free Immune-Conditioned Products in Advanced Solid Tumors: A Source-Aware Translational Framework
Source: Medicina (Kaunas). 2026 Oct 11;62(10):1964. doi: 10.3390/medicina62101964 (PMC13706797; doi:10.3390/medicina62101964)
Supplement: Supplementary file 1 [file medicina-62-01964-s001.zip › medicina-4519659-supplementary.pdf]

Supplementary Materials

Reproducible Search Strategy and Evidence Map

Cell-Free Immune-Conditioned Products in Advanced Solid Tumors: A Source-Aware Translational Framework

S1. Scope and Search Update

PubMed/MEDLINE searches were conducted without a date restriction and were updated through 2 August 2026. English-language records were prioritized; foundational consensus, methodological, regulatory, and reporting publications were retained when necessary to define a standard. The searches were supplemented by citation chaining and targeted retrieval from professional-society and regulatory repositories. The targeted update from 24 July through 2 August 2026 did not identify a source that materially altered the predefined framework; the final interpretive corpus therefore remained at 71 references.

These Supplementary Materials support a structured critical review rather than a systematic or scoping review. The blocks below are reproducible concept searches that can be rerun independently. The review preserves distinct evidence layers for product identity, biological activity, route feasibility, clinical observation, and comparative validation and does not pool treatment effects.

Table S1. Reproducible PubMed/MEDLINE search blocks.

Domain	Search string
Population	(("advanced solid tumor"[Title/Abstract] OR "metastatic cancer"[Title/Abstract] OR "refractory cancer"[Title/Abstract]) AND ("performance status"[Title/Abstract] OR frailty[Title/Abstract] OR "trial eligibility"[Title/Abstract] OR "treatment ineligible"[Title/Abstract]))
Host-directed biology	(("host-directed"[Title/Abstract] OR "immune modulation"[Title/Abstract] OR immunobiology[Title/Abstract] OR "immune engagement"[Title/Abstract]) AND (cancer[Title/Abstract] OR tumor[Title/Abstract]))
Source architecture	((secretome[Title/Abstract] OR "conditioned medium"[Title/Abstract] OR "extracellular vesicle"[Title/Abstract] OR "plant-derived"[Title/Abstract] OR phytosome[Title/Abstract] OR "milk-derived"[Title/Abstract] OR microbial[Title/Abstract] OR bacterial[Title/Abstract] OR fermentation[Title/Abstract] OR recombinant[Title/Abstract] OR synthetic[Title/Abstract] OR nanodisc[Title/Abstract]) AND (lipid[Title/Abstract] OR protein[Title/Abstract] OR matrix[Title/Abstract] OR assembly[Title/Abstract]))
Product class	(("cell-free"[Title/Abstract] OR secretome[Title/Abstract] OR "extracellular vesicle"[Title/Abstract] OR exosome[Title/Abstract] OR phospholipoprote*[Title/Abstract] OR "lipid-protein"[Title/Abstract]) AND (product[Title/Abstract] OR therapy[Title/Abstract] OR formulation[Title/Abstract]))
Product quality	(("potency assay"[Title/Abstract] OR identity[Title/Abstract] OR purity[Title/Abstract] OR "lot comparability"[Title/Abstract] OR stability[Title/Abstract]) AND (biologic[Title/Abstract] OR secretome[Title/Abstract] OR "extracellular vesicle"[Title/Abstract] OR "cell therapy"[Title/Abstract]))
Microdomains	(("membrane microdomain"[Title/Abstract] OR "lipid raft"[Title/Abstract] OR "immune synapse"[Title/Abstract] OR "microdomain reconstruction"[Title/Abstract] OR "membrane order"[Title/Abstract] OR "receptor clustering"[Title/Abstract]) AND (dendritic[Title/Abstract] OR immune[Title/Abstract]) AND (cancer[Title/Abstract] OR tumor[Title/Abstract]))
Intradermal route	((intradermal[Title/Abstract] OR "cutaneous delivery"[Title/Abstract] OR microneedle[Title/Abstract]) AND (immune[Title/Abstract] OR vaccine[Title/Abstract] OR "dendritic cell"[Title/Abstract] OR cancer[Title/Abstract]))
NAMs	(("new approach method*"[Title/Abstract] OR organoid[Title/Abstract] OR "microphysiological system"[Title/Abstract] OR "organ-on-chip"[Title/Abstract] OR "human-relevant"[Title/Abstract]) AND (nonclinical[Title/Abstract] OR safety[Title/Abstract] OR drug[Title/Abstract] OR biologic[Title/Abstract]))
Outcomes	((RECIST[Title/Abstract] OR iRECIST[Title/Abstract] OR PERCIST[Title/Abstract] OR

	SULpeak[Title/Abstract] OR "PET response"[Title/Abstract] OR "performance status"[Title/Abstract] OR "patient-reported outcome"[Title/Abstract] OR "biological engagement"[Title/Abstract]) AND immunotherapy[Title/Abstract])

S2. Evidence-Domain Map

Table S2 maps the final 71-reference interpretive corpus to the predefined evidence domains used in the main manuscript. Reference numbers correspond to the final main-manuscript bibliography.

Table S2. Evidence map of the 71-reference interpretive corpus.

Evidence domain	References	Primary contribution
Eligibility and representativeness	8, 12–14, 41	Performance-status restrictions, generalizability, trial eligibility, and broader inclusion.
Skin and intradermal immunobiology	4–5, 38–40	Cutaneous antigen-presenting cells, lymphatic access, vaccination rationale, and delivery feasibility.
Review method and host-directed rationale	9–11, 25–26, 28–29, 34	Narrative-review quality, structured synthesis, dendritic-cell and immune biology, immune signaling, and mechanistic boundaries.
Source and product taxonomy	1–3, 15–21, 31	MISEV terminology; source distinctions across human, non-human, plant, microbial, recombinant, and synthetic systems; composition-led naming; and source-aware product classification.
Characterization, potency, and platform examples	3, 6–7, 18, 22–24, 27, 32, 35, 46–47, 50, 54, 60, 69	Identity, potency, secretome reproducibility, immune-fingerprint preservation, proteomics, kinetic profiling, immune monitoring, traceability, lot comparability, and processed-product stability across the published evidence lineage.
Microdomain mechanisms and stability	6, 28–34, 36–37	Membrane organization, lipid rafts, receptor and immune-synapse organization, structural mechanism, extracellular lipid–protein architecture, and processed-product stability.
NAMs and nonclinical modernization	45–49, 51, 70–71	Context of use, scientific confidence, human-relevant nonclinical methods, regulatory modernization, and reduction or replacement of selected animal studies.
Function and patient-reported outcomes	14, 42, 55–56, 59	ECOG/performance status, supportive and palliative context, patient-reported outcomes, symptom deterioration, and longitudinal estimand definition.
Metabolic and structural imaging	57–58	RECIST/iRECIST-related interpretation, FDG PET/CT response assessment, metabolic response, atypical immune-response patterns, and pseudoprogression.
Clinical-development, real-world, and causal methodology	59, 61–65	Estimands, early-phase oncology development, Bayesian methods, comparative interpretation, target-trial emulation, causal inference, and TARGET reporting.
Reporting, safety, and ethics	42–44, 52–53, 66–68	Supportive-care governance, research ethics, Declaration of Helsinki, SPIRIT 2025, CTCAE, CONSORT 2025, extracellular-product safety, and conflict-of-interest disclosure.

S3. Published product-development evidence-lineage map

The seven publications are treated as complementary layers within one connected scientific-development lineage. They are not counted as seven independent demonstrations of clinical efficacy. Each row below preserves the evidence layer actually reported and the translational output that can legitimately be carried forward.

Table S3. Published evidence lineage of dendritic-cell-conditioned extracellular lipid–protein preparations.

Ref.	Published evidence layer	Principal reported contribution	Translational output
3	Platform definition	Introduces a dendritic-secretome-derived cell-free lipid–protein preparation and integrates analytical and functional characterization.	Establishes experimental tractability and a source-to-product starting point.
50	Immune-fingerprint preservation	Reports a proteomic profile exceeding 400 proteins with retention of more than 90% of immune-relevant markers after cryoprocessing.	Supports process resilience, identity panels, and comparability planning.
7	Monitoring architecture	Organizes product quality, flow cytometry, cytokine profiling, and longitudinal immune assessment.	Informs Module A biological engagement and integrated monitoring.
46	Real-time functional profiling	Uses label-free kinetic imaging to resolve non-cytotoxic phenotypic divergence across tumor-cell models.	Provides a NAM-aligned functional-stratification module.
47	Ex vivo traceability	Links formulation exposure, kinetic observation, and audit-ready documentation without clinical exposure.	Supports potency development, provenance, and reproducible decision records.
6	Mechanistic synthesis	Formalizes membrane microdomain organization, receptor clustering, immune-synapse geometry, and falsifiable repair hypotheses.	Provides the sequential microdomain evidence chain and perturbation/rescue agenda.
32	Processed-product stability	Documents reproducible proteomic features after concentration, cryopreservation, and lyophilization with short-term cellular compatibility.	Supports stability, comparability, and mechanism-linked qualification priorities.

S4. Update and Reproducibility Procedure

Before revision or resubmission, rerun all concept blocks from the last search date; screen newly identified records for material impact on the framework; verify DOI, publication year, journal, and article status against PubMed or the publisher; record additions and exclusions; update all in-text citations and the main bibliography; and regenerate Tables S2 and S3 so that every listed reference resolves to a cited source and each platform-specific statement remains aligned with its reported evidence layer.

Conversion of this structured critical review into a systematic or scoping review would require a protocol appropriate to that design, duplicate screening, a complete selection flow diagram, and method-appropriate appraisal tools. No such conversion is claimed in the present submission.
